# Agile software development one year into the COVID-19 pandemic

**DOI:** 10.1007/s10664-022-10176-9

**Published:** 2022-06-22

**Authors:** Pernilla Ågren, Eli Knoph, Richard Berntsson Svensson

**Affiliations:** 1 Chalmers University of Technology, Gothenburg, Sweden; 2 Chalmers | University of Gothenburg, Gothenburg, Sweden

**Keywords:** COVID-19, Pandemic, Remote work, Working from home, Agile software development, Bayesian analysis, Explanatory sequential mixed methods

## Abstract

As a result of the COVID-19 pandemic, many agile practitioners had to transition into a remote work environment. Despite remote work not being a new concept for agile software practitioners, the forced or recommended nature of remote work is new. This study investigates how the involuntary shift to remote work and how social restrictions imposed by the COVID-19 pandemic have affected agile software development (ASD), and how agile practitioners have been affected in terms of ways of working. An explanatory sequential mixed methods study was performed. Data were collected one year into the COVID-19 pandemic through a questionnaire with 96 respondents and in-depth semi-structured interviews with seven practitioners from seven different companies. Data were analyzed through Bayesian analysis and thematic analysis. The results show, in general, that the aspects of ASD that have been the most affected is communication and social interactions, while technical work aspects have not experienced the same changes. Moreover, feeling forced to work remotely has a significant impact on different aspects of ASD, e.g., productivity and communication, and industry practitioners’ employment of agile development and ways of working have primarily been affected by the lack of social interaction and the shift to digital communication. The results also suggest that there may be a group maturing debt when teams do go back into office, as digital communication and the lack of psychological safety stand in the way for practitioners’ ability to have sensitive discussions and progress as a team in a remote setting.

## Introduction

At the end of 2019 it was reported that a new virus had emerged (World Health Organization 2020c), and soon the whole world was faced with the consequences of what in March 2020 was officially declared by World Health Organization (WHO) to be the COVID-19 pandemic (World Health Organization 2020a). As the coronavirus spread across the world, more and more employees were asked to work from home as a measure to prevent and limit the spread. Several technology companies encouraged their employees to work remotely (Duffy 2020) and only a couple of months after the outbreak, WHO advised organizations to promote remote work as a measure to both keep the business going and ensuring peoples’ health (World Health Organization 2020b).

Working from home and regular remote work are not new phenomenons within software development. Several studies have compared distributed with co-located development (e.g., Devanbu et al. 2009), studies have investigated working from home and companies have adopted different working from home approaches with pre-existing polices (Nolan et al. 2021), and several studies have investigated regular remote work (Ralph et al. 2020). However, working from home and regular remote work are not the same as working remotely during the COVID-19 pandemic (Ford et al. 2020; Ralph et al. 2020; Bezerra et al. 2020; Miller et al. 2021; Silveira et al. 2021). Instead, practitioners and entire software development teams that used to work predominantly in-person were, unexpectedly overnight, forced to change from mostly co-located to remote work (Miller et al. 2021). This involuntary and rapid shift makes working remotely during the COVID-19 pandemic different from regular remote work. Additional challenges include lack of proper infrastructure (including physical infrastructure), the need to care for children, and fear of COVID-19 (Silveira et al. 2021). Another difference between regular remote work and working remotely during the COVID-19 pandemic is the social restrictions.

While numerous studies have investigated working from home and regular remote work, only some have investigated the COVID-19 pandemic’s impact on software development, and few have investigated COVID-19 and ASD. Previous studies have investigated software developers’ and software engineers’ productivity and well-being with contradictory results (e.g. Bao et al. 2020; Ralph et al. 2020; Ford et al. 2020), others (e.g. Russo et al. 2021a) found that well-being and productivity are correlated, while Russo et al. (2021b) found that except for a correlation between productivity and breaks, there is no significant effect between working activities (e.g., coding, testing, code review, and meetings) and well-being or productivity. A few studies (e.g. da Camara et al. 2020; Marek et al. 2021; Schmidtner et al. 2021) focused on industry practitioners in ASD in particular, where Marek et al. (2021) found that remote work during the COVID-19 pandemic does not seem to affect agile teams’ productivity.

Most of the previous studies related to the COVID-19 pandemic and software development have been conducted in the early stages of the pandemic. The first few months may have been a time of confusion and uncertainties about and adjustments to the new situation, especially when working from home. Several things may happen within one year, the sudden shock and adjustment period may have disappeared and left more unstructured processes regarding remote work behind. Therefore, this study focuses specifically on how ASD have been affected by the COVID-19 pandemic, by conducting the study approximately one year after the beginning of the COVID-19 pandemic. In addition, while other studies mention a “forced” aspect of the current working from home situation, also called “involuntary, remote work” (Bezerra et al. 2020) or “mandatory remote work” (Machado et al. 2021), none has investigated what implications the feeling of involuntary and forced remote work has on industry practitioners, which is investigated in this study.

In this paper, we investigate how the involuntary shift to remote work and how social restrictions imposed by the COVID-19 pandemic have affected ASD, its practitioners, and their ways of working. To this aim, we conducted an explanatory sequential mixed methods study consisting of a quantitative and a qualitative phase. Data was collected approximately one year into the pandemic, between February and April 2021, through a questionnaire with 96 respondents and in-depth semi-structured interviews with seven practitioners from seven different companies. The results of this study show that communication and social interactions have been affected the most, while technical practices have not experienced the same changes. Moreover, stand up meeting, retrospective, and pair programming have been three especially important agile practices during the COVID-19 pandemic since they have been used to address the lack of social interaction. Feeling forced to work remotely has had a significant impact on, e.g., productivity, well-being, communication, and meetings. Meaning, a lower productivity, well-being, communication quality, and a higher meeting frequency are all examples of significant effects for a practitioner who feels forced to work remotely.

The remainder of this paper is organized as follows. Section 2 presents related work. Section 3 describes the design of our mixed method study. Section 4 presents the statistical analysis and the results, while the results are discussed in Section 5. Section 6 discloses the threats to the validity of our study. Finally, Section 7 gives a summary of the main conclusions.

## Related Work

Several papers have studied the COVID-19 pandemic’s impact on software development (Ford et al. 2020; Bao et al. 2020; Ralph et al. 2020; Russo et al.2021a, 2021b; Machado et al. 2021; Silveira et al. 2021; Miller et al. 2021; Rodeghero et al. 2021). Ford et al. (2020) conducted a survey with 3646 Microsoft employees to understand the effects of remote work due to the pandemic on employees’ well-being and productivity. The results show no significant evidence that the employees’ productivity has been substantially affected by the pandemic. However, the results show that the experience differs between employees, what one employee sees as a benefit the other might see as a disadvantage. For example, employees that like to work from home due to the freedom reported a higher productivity, whereas employees who missed the office reported a lower productivity. The same dichotomous experience applies to, e.g., well-being, ability to focus, work environment, and meetings. Bao et al. (2020) compared 139 developers’ activity records before and after the COVID-19 pandemic to measure the developers’ productivity. The results show that larger projects and the type of projects are significant factors to why a developer might feel less productive now than before the pandemic (Bao et al. 2020). Ralph et al. (2020) conducted a survey with 2225 software developers from many different domains all around the world to understand the effects of the COVID-19 pandemic on developers’ work environment, productivity, and well-being. The results show that the COVID-19 pandemic has had a negative impact on developer’s productivity and well-being (Ralph et al. 2020). Moreover, Ralph et al. (2020) concluded that women, parents, and people with disabilities may be dis-proportionality affected by the COVID-19 pandemic. A similar survey with 233 respondents in Brazil investigated the COVID-19 pandemic’s impact on gender inequality (Machado et al. 2021). The results suggest that most organizational incentives adopted to facilitate remote work were dis-proportionality benefiting men (Machado et al. 2021). Even though women and men reported similar levels of interruptions, they experienced different types of interruptions and effects on their well-being (Machado et al. 2021).

Russo et al. (2021b) conducted two surveys with 192 software engineers, one in April 2020 and one in May 2020, to understand what a normal day in a software engineer’s work life looks like during the COVID-19 pandemic, and how and what activities have an impact on their well-being and productivity. The results show that software engineers spend about the same time on activities at home as they did onsite. However, the software engineers spend less time in meetings and breaks, and there is no significant relation between well-being and productivity (Russo et al. 2021b). In another study (Russo et al. 2021a), the results suggest that software engineers have adopted to the COVID-19 pandemic over time, and thus working from home is not a significant challenge. In addition, the study shows that, on average, the software engineers’ well-being has increased during the COVID-19 pandemic, and that there is a correlation between well-being and productivity (Russo et al. 2021a). In Silveira et al. (2021), the authors investigated the impact of COVID-19 on daily activities and well-being by conducting a mining software repository study with 100 GitHub projects developed in Java, which was complemented by a survey with 279 software development professionals. The findings in Silveira et al. (2021) are similar to other studies, i.e., the COVID-19 pandemic’s impact is a spectrum and not a matter of just an increase or decrease in, e.g., productivity.

Miller et al. (2021) explored how software developers have been affected by working from home (WFH) during the COVID-19 pandemic. They investigated changes in teams’ ability to meet milestones, team culture, support, communication, collaboration, social interactions, and what factors that are related to changes in team productivity by conducting a qualitative and a quantitative survey with 2265 and 608 responses respectively (Miller et al. 2021). The results show that social connection and communication are two of the most prominent challenges experienced. In addition, most of the respondents experienced little or no change in team productivity. However, the reported productivity is in a wide spectrum, with almost as many reporting an increase as decrease. Other findings include, developers experiencing having more meetings, an increased empathy and understanding for each others’ situation, the fostering of social interaction through social engagement meetings, and having personal check-ins to examine well-being and progress. Another study (Rodeghero et al. 2021) conducted a survey with 267 new software developers followed by eight interviews to investigate the remote onboarding experiences. The study presents three main findings, the developers faced many challenges, some unique to the special WFH situation and others were not; although teams had social events to connect with new hires, some new hires did not felt socially connected; and newly hired software developers want more manager meetings and overall interaction with their teams.

While none of the aforementioned studies have primarily focused on COVID-19 and ASD, a few others have (da Camara et al. 2020; Marek et al. 2021; Schmidtner et al. 2021; Neumann et al. 2021). An action study investigated how startups handle remote work and what agile practices and activities are useful during the COVID-19 pandemic (da Camara et al. 2020). To reduce uncertainties during the COVID-19 pandemic, the study found that it is important to conduct socialization events and guidelines, but also to establish knowledge sharing rounds and code development standards. In another study, based on a survey with 120 respondents, the results show that ASD teams’ work was not significantly affected by the COVID-19 pandemic (Marek et al. 2021). The ASD teams transitioned to remote work without any major issues because many of the teams already used remote working tools before the pandemic. In addition, the results show that unnecessary meetings were reduced, productivity increased because of remote work, and that ASD teams that had a mix of on-site and distributed team members improved their communication when all team members were working remotely (Marek et al. 2021). Schmidtner et al. (2021) conducted a study in Germany focusing on managers and project management experts in agile development. The study found that the “lockdown” had a significant impact on agile working. The results show that the transition to remote work went smoothly, practitioners now use online tools that they were reluctant to use before the pandemic, experience an increase in flexibility, with only a small loss in productivity (Schmidtner et al. 2021). Another study, also conducted in Germany, observed three ASD teams and their use of agile practices during the COVID-19 pandemic (Neumann et al. 2021). The study found, in all three ASD teams, that pair programming, retrospectives, and daily stand-up meetings, among others, were used. Moreover, the study also found that daily, instead of weekly, stand-up meetings were conducted to increase synchronization in the team. In addition, pair programming was found to be conducted in several different ways using different online tools.

## Research Method

The aim of this study was to investigate the COVID-19 pandemic’s impact on Agile Software Development (ASD). This aim was achieved by addressing the following research questions (RQ):

- **RQ1:** Compared to before the pandemic, how has the way industry practitioners employ agile development and ways of working changed?
- **RQ2:** How has industry practitioners been affected by recommended or enforced remote work?

**Fig. 1:**
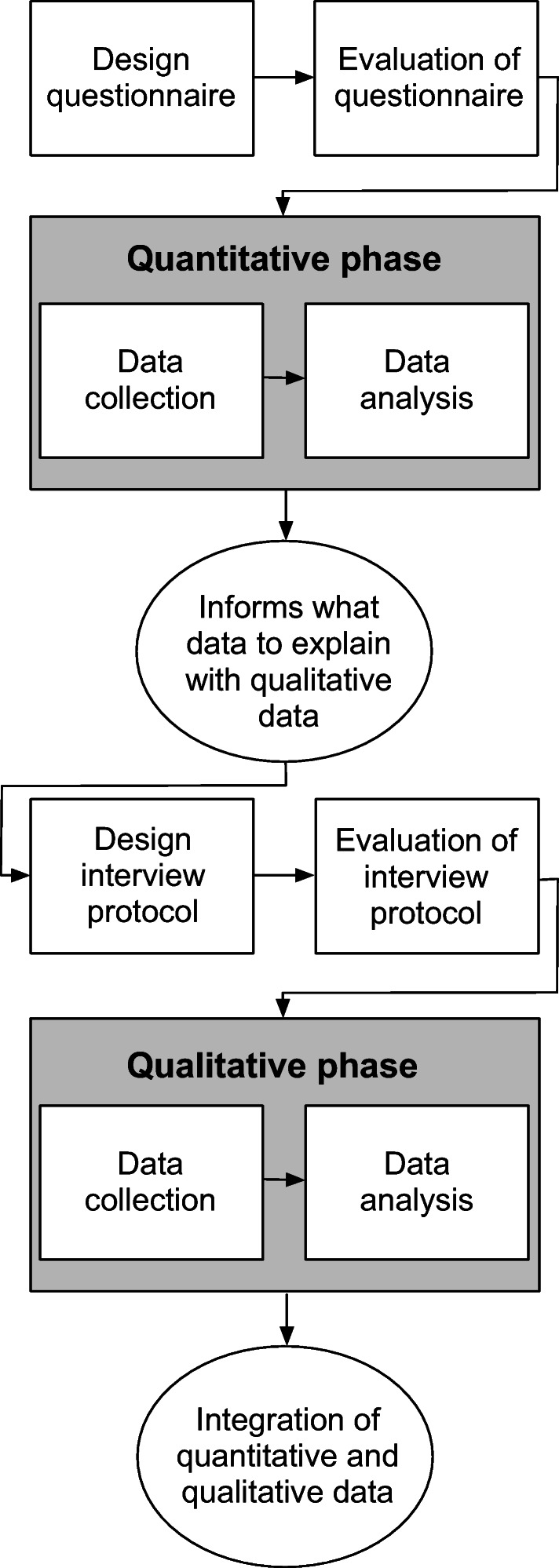
Overview of the study design

A quantitative approach, such as a questionnaire, would not be able to capture the reasoning behind the answers, whereas a qualitative approach, such as an interview, would not be sufficient for providing a general description of the situation. However, combining both these approaches would achieve a better understanding of the COVID-19 pandemic’s impact on ASD compared to using a quantitative or qualitative research approach alone (Creswell 2015). Therefore, the investigation presented in this paper was carried out using an explanatory sequential mixed methods approach (Creswell 2015) since it would best meet the objectives of this study. The study consisted of a quantitative phase (a questionnaire) followed by a qualitative phase (interviews), which is illustrated in Fig. 1, and described in more details below.

### Quantitative Phase

The quantitative phase consisted of a questionnaire which is time effective, easily administrated, and provides an opportunity to collect data from a large number of respondents from different geographical locations (Singer et al. 2008). In addition, questionnaires enable automation in the data collection phase (Easterbrook et al. 2008). Given the aim of this study, a questionnaire was a suitable approach since we could begin with a broad overview based on a diverse set of respondents.

#### Questionnaire Design

We designed a self-administered cross-sectional web-based questionnaire (Kitchenham et al. 2008) using QuestionPro’s survey tool.^1^ The questionnaire was designed with respect to the different areas of interest, inspiration from previous studies (Ralph et al. 2020; Ford et al. 2020; Russo et al. 2021b), and a pre-study.

A pre-study was conducted since it can be difficult to directly convert objectives to questions (Kitchenham et al. 2008). The pre-study consisted of three open-ended questions regarding agile development and the COVID-19 pandemic. The sampling strategy used for the pre-study was convenience sampling (Runeson and Höst 2009) within our industrial network. Five agile practitioners participated in the pre-study. The questions were directed to practitioners’ own experiences and opinions regarding changes and perceptions due of the COVID-19 pandemic. The feedback generated a few additional closed-ended questions but also justified having the open-ended questions we already had.

The questionnaire consisted of a mix of closed-ended and open-ended (free text) questions. The closed-ended questions were of the following types: select one option, Likert scales, and ordinal scales. Most of the questions followed the same structure, and were introduced as “Compared to before the pandemic...” to capture the level of change experienced. Most questions included either an ‘Other’ option or a ‘Non applicable’ (N/A) option. The final version of the questionnaire^2^ was divided into nine sections containing 28 questions (referred to as Q1–Q28).

**Fig. 2:**
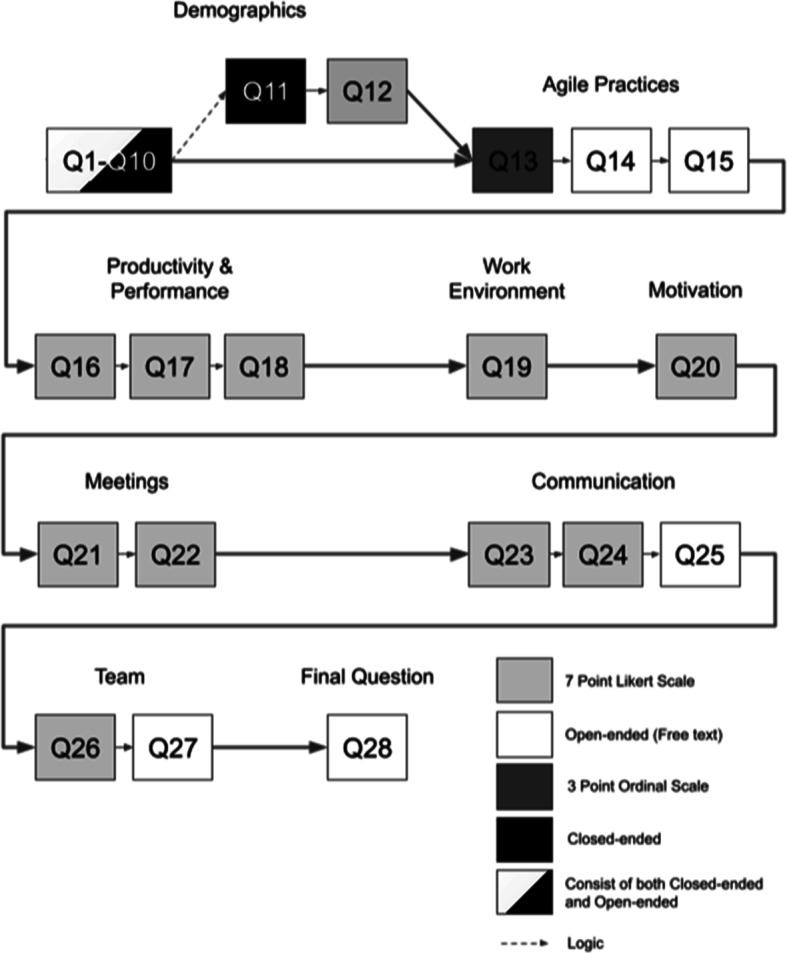
Overview of the questionnaire

Figure 2 provides an overview of the questionnaire. The demographic section covered questions regarding, e.g., team size (Q2), domain (Q4), role (Q6), and experience with ASD (Q7). In addition, it also covered to what extent the respondents had been working remote before (Q9) and during the COVID-19 pandemic (Q10). Those who had been working remote during the pandemic were asked to answer why they are working remote (Q11) and whether they felt forced to do so (Q12). Thus, Q11–Q12 were included to capture how involuntary the shift to remote work was experienced.

The next section of the questionnaire (Q13–Q15) investigated how the deployment of agile practices had been affected by the COVID-19 pandemic. Since “agile work practices are harder to perform” (Mancl and Fraser 2020, p. 310) in distributed agile environment caused by the COVID19-pandemic, it was important to investigate this. Q13 covered how challenging 19 common agile practices are now, compared to before the pandemic. The list of the 19 common agile practices was based on cross-referencing Agile Alliance’s list of 56 agile practices (AgileAllliance 2020), Version One’s 14th Annual State of Agile report’s list on the most used agile practices and techniques of 2020 (Digitalai 2020), and a systematic literature review summarizing agile practices used in global software development (Vallon et al. 2018). As the list did not consist of all existing agile practices, Q14-Q15 enabled the respondents to add information on any practice, from the list or not, that they had started to use or abandoned (Q14), or considered particularly important (Q15) during the COVID-19 pandemic. The productivity and performance section (Q16–Q18) covered self-assessed productivity on both individual (Q16) and team level (Q17); perception of quality of work, availability, focus, distractions, interruptions, and customer satisfaction (Q17); and potential changes in time spent on certain activities (Q18). The work environment section contained one question (Q19) about e.g. the respondents’ well-being, work/life balance, and work environment. Motivation is highlighted in the agile principle “build projects around motivated individuals” (Beck et al. 2001b), but is, to the best of our knowledge, not as prevalent as other factors in previous COVID-19 studies. Therefore, Q20 covered statements in terms of pure motivation, feeling of being overwhelmed, ability to choose and focus on a given task, acknowledgement of work, and perception of having a meaningful work. Q21-Q22 covered different aspects of meetings, where Q21 measured type of change in terms of increase/decrease, whereas Q22 compared how often (frequency) specific meetings occur now compared to before the pandemic. The communication section (Q23–Q25), dealt with how industry practitioners’ communication within and outside of teams has changed due to the COVID-19 pandemic. As agile teams are a central part of the agile methodology, and due the current situation (the COVID-19 pandemic), the questionnaire also included questions (Q26-Q27) regarding team inclusion, team morale, and transparency. Finally, the questionnaire was concluded by a final question (Q28), which was an open-ended question where the respondents had the opportunity to add anything related to how the COVID-19 pandemic has affected ASD. Table 1 provides an overview of how the questions in the questionnaire are linked to the two research questions.

**Table 1:** Link between the questionnaire and research questions

Questionnaire section	Questionnaire question(s)	Research question(s)
Demographics	Q1–Q8	–
	Q9–Q12	RQ2
Agile practices	Q13–Q15	RQ1
Productivity & Performance	Q16, Q17	RQ2
	Q18	RQ1
Work Environment	Q19	RQ2
Motivation	Q20	RQ2
Meetings	Q21	RQ1
	Q22	RQ1, RQ2
Communication	Q23–Q25	RQ1
Team	Q26, Q27	RQ1
Final Question	Q28	–

#### Questionnaire Evaluation

We evaluated the questionnaire in two steps. In the first step, the authors reviewed the questions with focus on content, clarity, and connection to the research questions. In addition, we invited a software engineer to review the questionnaire regarding clarity and wording. The feedback from the software engineer was that the questions were understandable and clear. In the second step, we invited two agile practitioners and one software engineering student to take part in a pilot study. We asked the pilot respondents to measure the time needed to complete the questionnaire, and to provide feedback on the clarity and content of the questionnaire, the used scales, and if there was anything that they missed or would like to add. The feedback from the pilot respondents led to clarification of Q13, increased font size for subheadings and instructions, clarification of whether the alternative “other” was of the free-text type for questions with many alternatives, and to highlight that the time referred to as “now” corresponds to “now, during the pandemic”. One pilot respondent voiced a wish for one additional question. All of these aspects were taken into account and adjusted for in the final version of the questionnaire, which is presented in Fig. 2.

#### Data Collection

Our target respondents were industry practitioners involved in ASD, with the inclusion criteria (Kitchenham et al. 2008) that they had been working both before and during the COVID-19 pandemic. Respondents who did not apply to these criteria were filtered out in the demographics section. The sampling techniques used for the questionnaire were convenience sampling (Kitchenham et al. 2008), maximum variation sampling (Etikan et al. 2016), and snowball sampling (Kitchenham et al. 2008).

Data collection started on 10 February 2021 and finished on 15 March 2021. The authors directly contacted industry practitioners with different roles, experiences, and from different domains, and asked those industry partners to distribute the questionnaire within their organizations. Moreover, the questionnaire was distributed to industry collaboration networks and communities dedicated to ASD on LinkedIn, Facebook and Reddit.

#### Data Analysis

In general, the software engineering discipline handles ordered categorical (ordinal) data by assuming that the conclusions are not depending on if a regression or ordinal model is used. The problem with this assumption is, that relying on an incorrect outcome distribution will lead to subpar predictive capabilities of the model (Bürkner and Vuorre 2019). Other problems include that effect size estimates will be biased when averaging multiple ordinal items, and that the data can be non-normal. This is something a researcher should handle (Liddell and Kruschke 2018) in a principle way, which we can do in Bayesian data analysis. Furthermore, limitations of frequentist statistics, such as the arbitrary *α* = 0.05 cut-off, significance testing of null hypothesis, and the reliance on confidence intervals, have been criticized (Ioannidis 2005; Morey et al. 2016; Nuzzo 2014; Woolston 2015). There are several advantages of using Bayesian statistics over frequentist statistics, e.g., to provide nuanced analyses and to improve generality and predictive ability (Furia et al. 2019). Bayesian statistics have the potential to provide more informative results than frequentist statistics since Bayesian statistics are based on probability distributions that are more informative than the single point estimates with approximate measures of uncertainty that are used in frequentist statistics (Furia et al. 2019; Bürkner and Vuorre 2019).

Therefore, in this study, we used a Bayesian statistical approach using ordinal Bayesian regression models (Bürkner and Vuorre 2019) for analyzing quantitative data, where the dependent variable is referred to as an outcome or response variable, and the independent variables referred to as predictors. The outcomes are based on Likert-item^3^ data from a questionnaire, which means that the outcomes are of an ordered categorical type. For ordered categorical effects, the difference is not necessarily the same between all categories (Bürkner and Vuorre 2019). That is, for a seven-point Likert scale ranging from strongly disagree to strongly agree, where strongly disagree is 1, disagree 2, agree 6, and strongly agree 7, the difference between 1 and 2 is not necessarily equal to the difference between 6 and 7. When using ordinal regression models, there are at least three ordinal model classes to choose from, Cumulative (Walker and Duncan 1967), Sequential (Tutz 1990), and Adjacent-Category (Bürkner and Vuorre 2019). As a rule of thumb, the cumulative model is often a suitable choice for Likert-item data sets (Bürkner and Vuorre 2019), which is the case of this study.

##### Data Cleaning

We started the analysis by filtering out invalid answers, i.e., empty responses and respondents who only answered the demographic questions. Since the COVID-19 pandemic had been going on for more than a year (at the time when the questionnaire was sent out), respondents were required to have worked for at least one year and been using agile methodologies. Responses who did not fulfill these criteria were also removed. In total, 155 responses were obtained, of which 75 were complete (48%). Partial responses that fulfilled the above criteria were kept, resulting in 21 remaining responses. Thus, the cleaned data set consisted of 96 responses.

##### Model Preparation

When preparing for the statistical model development, 11 out of 12 demographic questions were set as predictors (Q1-Q7, Q9–Q12). Q8 (which development methodology do you mainly follow in your team/work now?) was excluded from the data analysis since the data cleaning described above excluded practitioners who do not follow an agile methodology. The remaining closed-ended questions (Q13, Q16–Q24, and Q26) were set as outcomes. Table 2 provides a short description of the predictors. In order to facilitate the analysis, some categories (with few or no responses) were regrouped. For example, role (Q6) had 22 categories and was regrouped to ‘Technical’ (e.g, developer and software engineer), ‘Management’ (e.g., product manager and project leader), and ‘Agile’ (e.g., scrum master and product owner). The final regrouping is shown in Table 2. After gaining some insight concerning the data and the predictors, we next turn our attention to statistical model development where we design, compare, validate, and diagnose statistical models to conduct inferences.

**Table 2:** Predictors of interest

ID	Predictor name	Value(s)	Type
P_Q1_3C	Gender	female (1), male (2), other (3)	ℤ
P_Q2_s	Team size	0,...,41	ℕ
P_Q3_5C	Team constellation	exactly same (1), mostly same (2), changed team but same employment (3), changed employment (4), not part of a team (5)	ℤ
P_Q4_3C	Domain	IT (1), Embedded Systems (2), Others (3)	ℤ
P_Q5_3C	Continent	Europe (1) , North America (2) , Asia (3)	ℤ
P_Q6_3C	Role	Technical (1), Management (2), Agile (3)	ℤ
P_Q7_s	Years of experience	0,...,30	ℕ
moP_Q9_1_5L	Remote work before	never remotely (1),..., never at office (5)	O
moP_Q10_1_5L	Remote work now	never remotely (1),..., never at office (5)	O
P_Q11_7C	Reason	Recommendation from company (1), government (2), company & government (3); Enforcement by company (4), government (5), company & government (6); My own choice (7)	ℤ
moP_Q12_1_7L	Forced	strongly disagree (1),..., strongly agree (7)	O

From left to right, ID (encoding used in our model), predictor name, the values that can be used, and type. For type, ℕ indicates a natural number, O indicates ordered (categorical) data, and ℤ indicates an integer for categorical data

##### Statistical Model Development

The statistical models were designed using the brms (Bayesian regression models using ‘Stan’) package (Bürkner and Vuorre 2019) in R since it can handle the three ordinal model classes Cumulative (Walker and Duncan 1967), Sequential (Tutz 1990), and Adjacent-Category (Bürkner and Vuorre 2019). The final models were designed on the form $O \sim \ p_{1} + p_{2} + ... + p_{12}$ where *O* represents an outcome and *p*_*n*_ represents a predictor. The predictors Q2 (team size) and Q7 (years of experience) were continuous variables, and were therefore standardized.^4^ The predictors Q9 (remote work before), Q10 (remote work now), and Q12 (forced) were ordered categorical predictors, and were modeled with monoticity, which means that the variance between the different categories may differ (Bürkner and Vuorre 2019). In total, 100 models for the outcome variables were desgined, and one model was also designed for the predictor Q12 (forced) since Q12 could serve as an outcome dependent of the other predictors.

**Fig. 3:**
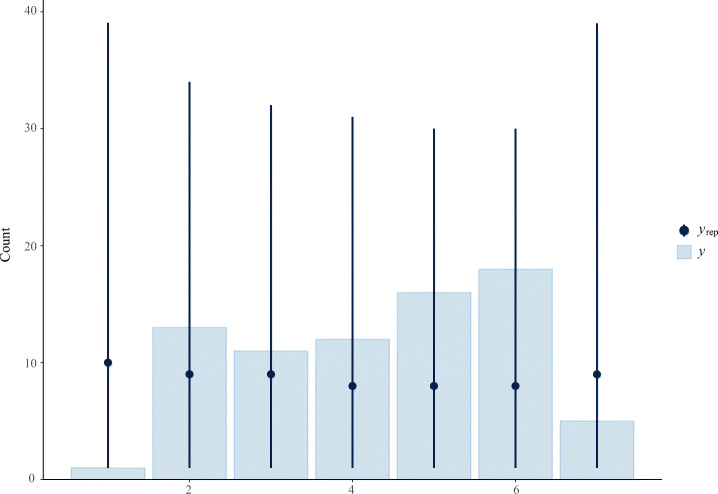
A Prior Predictive Check of the model with Q16—self-assessed productivity—as outcome variable. Sampling only from priors

**Fig. 4:**
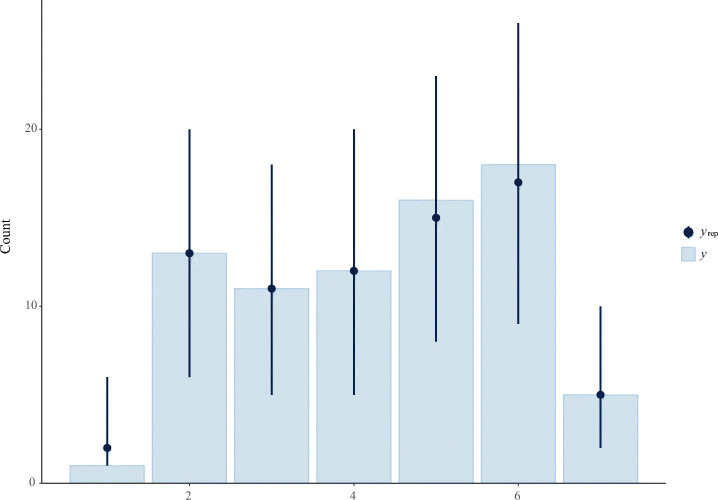
A Posterior Predictive Check of the model with Q16—self-assessed productivity—as outcome variable. Sampling from observed data
*Diagnostics*.: Hamiltonian Monte Carlo was used for checking the diagnostics of the models, which should be performed to ensure validity and efficiency of sampling. For our models, the used diagnostics were divergence, energy, and tree depth. There should be no divergences since this is an indication that the posterior may be biased, hence the model cannot be trusted (McElreath 2015). Having low energy values of the Bayesian fraction of missing information (E-BFMI) is also an indication that the posterior may be biased (Betancourt 2016). For efficiency concerns, the tree depth should be kept as small as possible. Reaching maximum tree depth indicates that the sampler is terminating prematurely to avoid long execution time. For our model, no divergences were reported, and no warnings were reported for neither E-BFMI nor tree depth.
  In addition, we checked the $\widehat {R}$ convergence diagnostics (McElreath 2015), which indicates if the independent chains converged, i.e., explored the posterior in approximately the same way. $\widehat {R}$ should go towards 1.00, but if it exceeds 1.01 it is an indication of bias. The $\widehat {R}$ diagnostics were consistently < 1.01. Finally, the effective sample size (ESS) (McElreath 2015), which captures how many independent draws contain the same amount of information as the dependent sample obtained for each parameter, was checked. ESS should not be below 0.1. The ESS diagnostics were consistently > 0.3.

*Selection of likelihood*.: The first step concerning model design is to decide which likelihood to use for inference, Cumulative, Sequential, or Adjacent-Category. To decide which likelihood to use, a model comparison was performed. The model comparison did not show any significant difference; thus, Cumulative was used since it is suitable for Likert-item data (Bürkner and Vuorre 2019). Next, we need to set appropriate priors.
*Prior and posterior predictive checks*.: For our models, we had several predictors in need of appropriate priors. A sensitivity analysis was performed to decide on priors to make sure that the combination of all priors are nearly uniform on the outcome scale (Furia et al. 2019). The complete model design, with priors, is thus,
  $$ \begin{array}{@{}rcl@{}} y_{i} & \sim& \mathsf{Cumulative}(\phi_{i}, \kappa) \\ \mathrm{logit(\phi_{i})} & = &\beta_{1} \times \mathrm{P\_Q1}_{i} + \beta_{2} \times \mathrm{P\_Q2}_{i} + \beta_{3} \times \mathrm{P\_Q3}_{i} + \beta_{4} \times \mathrm{P\_Q4}_{i} + \\ && \qquad \beta_{5} \times \mathrm{P\_Q5}_{i} + \beta_{6} \times \mathrm{P\_Q6}_{i} + \beta_{7} \times \mathrm{P\_Q7}_{i} + \beta_{9} \times \mathrm{mo(P\_Q9)}_{i} + \\ && \qquad \beta_{10} \times \mathrm{mo(P\_Q10)}_{i} + \beta_{11} \times \mathrm{P\_Q11}_{i} + \beta_{12} \times \mathrm{mo(P\_Q12)}_{i} \\ \beta_{1,...,7,11} & \sim& \text{Normal}(0, 0.25) \\ \beta_{9,10,12} & \sim& \text{Dirichlet}(2) \\ \kappa & \sim& \text{Normal}(0, 2) \end{array} $$
  The first line corresponds to the likelihood of the model. On lines 2–4 we provide our linear model with all predictors and parameters we want to estimate, where *β* is a prior, P_Q a predictor, and mo() the notion of a monotonic predictor. Finally, on lines 5–7, we set priors on our parameters. For most of the predictors, the priors were set to ‘Normal(0, 0.25)’,^5^ and for the ordered categorical (monotonic) predictors, the priors were set to ‘Dirichlet(2)’. The intercept (cutpoints) priors for *κ* were set to ‘Normal(0, 2)’.
  A visual view is given by sampling from the priors only, i.e., prior predictive checks, and with priors and data, i.e., posterior predictive checks. Figure 3 plots the prior predictive check, while Fig. 4 plots the posterior predictive check. The dots indicate the mean while the lines indicate the uncertainties. The bars represent the observed data. Looking at Fig. 3, we see that the means were approximately at the same level, and the uncertainties of each level were fairly uniform. In Fig. 4 we see that the model estimated perfectly on four levels, while the others were slightly above or below, which is acceptable.

### Qualitative Phase

In the qualitative phase, which took place after the analysis of the quantitative data, we conducted semi-structured interviews (Runeson and Höst 2009) with industry practitioners to explore and explain the quantitative data in more depth.

#### Interview Protocol Design

From the questionnaire data analysis, the aspect of feeling forced to work remotely or not (Q12) was a statistically significant predictor in 23 out of 29 outcome questions (see Section 4.2). Furthermore, there was at least one outcome with significant predictors in all questionnaire sections which justified having similar themes in the interview protocol. Based on the significant predictors, and by reviewing the responses to the open questions (Q14, Q15, Q25, Q27 and Q28) in the questionnaire, and considering the RQs, the interview protocol was designed.

As the forced aspect (Q12 in the questionnaire) seemed to be particularly important, it was important to investigate why some feel forced, and why some do not feel forced to work remote. Hence, the first part of the interview protocol (Q1–Q5) was dedicated to questions regarding the transition to remote work (Q1), whether they felt forced to work remotely (Q2), if their view has changed since the beginning of the pandemic (Q3), and pros and cons regarding the situation (Q4-Q5). The next section of the interview protocol (Q6–Q9) investigated how the deployment of agile practices had been affected by the COVID-19 pandemic. Q6 covered if any agile practices are less or more challenging now compared to before the pandemic. Q7–Q9 enabled the interviewee to add information about any practices they had started to use or abandoned since the pandemic (Q7), if any practice is more important now than before the pandemic (Q8), and what agile practices they use (Q9). The communication and meetings section (Q10–Q11) dealt with how the interviewees’ communication within and outside of teams has changed due to the COVID-19 pandemic (Q10), and changes in meetings (Q11). The productivity and performance section (Q12–Q13) covers self-assessed productivity (Q12) and work performance (Q13). The work environment section contained one questions (Q14) about changes in their work environment, while the team section contained one question (Q15) about any experienced differences in how their team worked now compared to before the pandemic.

#### Interview Protocol Evaluation

We evaluated the interview protocol by conducting a pilot study with one industry practitioner. The focus of the pilot study was to evaluate the quality and clarity of the questions, and the length of the interview. The feedback from the pilot study led to shortening the number of questions asked, and clarifications of some questions. All of these aspects were taken into account and adjusted for in the final version of the interview protocol.^6^

#### Data Collection

The sampling strategy used was convenience sampling (Runeson and Höst 2009) where four practitioners from our industrial network agreed to participate, two registered an interest in participating in interviews when answering the questionnaire, and one from a dedicated agile forum. Thus, seven practitioners from seven software developing companies participated. A characterization of the interviewees can be seen in Table 3.

**Table 3:** Interview subjects characteristics

ID	Role	Gender	Remote	Remote	Changed	Answered	Country
			before	now	team	questionnaire	
P1	Software Engineer	Male	0%	100%	Yes	Yes	Sweden
P2	Product Manager	Male	10%	100%	Yes	Yes	Sweden
P3	Software Developer Consultant	Male	0%	90%	No	No	Sweden
P4	Technical Agile Coach	Male	≈ 0%	100%	No	Yes	Sweden
P5	Software Developer Consultant	Female	0%	≈ 100%	Yes	Yes	Sweden
P6	Software Developer	Male	0%	100%	Yes	No	Sweden
P7	Software Developer	Male	0%	100%	Yes	No	UK

We conducted seven semi-structured interviews in April 2021. One interviewee and two interviewers (the first two authors where one took notes and one asked the questions) attended all interviews. During the interviews, which were held online in Zoom or Google Meet, the purpose of the study was presented to the interviewee. Then, questions about the different areas of interest were discussed in detail. For all interviews, varying in length from 30 to 45 min, we took records in the form of written extensive notes and audio recordings in order to facilitate and improve the analysis process.

#### Data Analysis

The qualitative data was analysed using the six phase thematic analysis process established by Braun and Clarke (2006), which includes: familiarizing yourself with your data, generating initial codes, searching for themes, reviewing themes, defining and naming themes, and producing the report.

In the first phase, familiarizing yourself with your data, the first two authors went through the notes from each interview and underlined the most interesting and important parts, and transcribed the audio recordings obtained from the interviews. Then, a summary of each interview was sent to the respective interviewee for validation of our interpretation. Six out of seven interviewees responded with feedback. One interviewee provided feedback regarding one aspect that we had misunderstood, which we changed accordingly. The other five interviewees responded that they were fine with the summaries of the interviews. All the transcribed data and notes from the interviews were imported into NVivo v.12,^7^ a software tool for analysing qualitative data.

The second phase, generating initial codes, followed the process of open coding. Open coding begins by systematically reading through the transcripts and notes. Statements from the transcribed interviews and the notes were read and coded by the first and second authors. Codes were assigned until no further codes were discovered. The collection and analysis of data was an iterative and interpretive process.

**Fig. 5:**
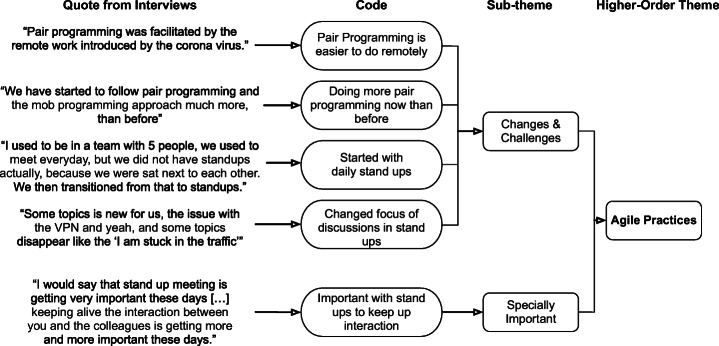
Example of the open and axial coding process

The third phase, searching for themes, followed the process of axial coding. All the codes that were created in the second phase were organized into potential themes. For example, the quote “Pair Programming is easier to do remotely” was added to the sub-theme Changes & Challenges of the higher-order theme Agile Practices, since it described a change in how challenging an agile practice was experienced, which is illustrated in Fig. 5.

Once we started reviewing themes (fourth phase) and their respective data extracts and codes, we found that some extracts were not forming a coherent pattern and did not fit into the potential themes. As a result, themes were renamed, merged, and new themes were created.

In the fifth phase, defining and naming themes, we defined and refined all themes, and conducted a detailed analysis of each theme as well as identified its narrative. At this stage, we ended up with a total of eight higher-order themes, which are further described in Section 4. Before moving on to the sixth phase, interviews were conducted until a point of theoretical saturation was reached.

In the sixth and final phase, producing the report, the details of these concepts together with a thematic map are explained in Section 4.

### Integration of Quantitative and Qualitative Data

There are different ways to integrate quantitative and qualitative data when conducting a mixed method study (Creswell 2015), e.g., at the time of the analysis, and/or during the data collection by, e.g., collecting both open-ended and closed-ended responses. For a mixed method study of a sequential explanatory design, initiated by a quantitative phase followed by a qualitative phase where the aim for the qualitative phase is to help to explain the quantitative data with qualitative data, the integration refers to the explanation of the data (Creswell 2015). In this study, there is an emphasis on the quantitative phase since the qualitative phase is used to support the interpretation and explanation of the quantitative data. Both significant and non-significant results from the Bayesian analysis were investigated before the interviews were conducted. By looking into the themes that emerged from the thematic analysis of the qualitative data, we found explanations for the quantitative data, both regarding significant results and descriptive statistics. In addition, the answers to the open-ended questions in the questionnaire served as an additional source that was used to explain the quantitative data.

## Results

This section presents the questionnaire respondent demographics, the analysis of the questionnaire, and then the results organized according to the research questions in Section 3.

### Questionnaire Respondent Demographics

**Fig. 6:**
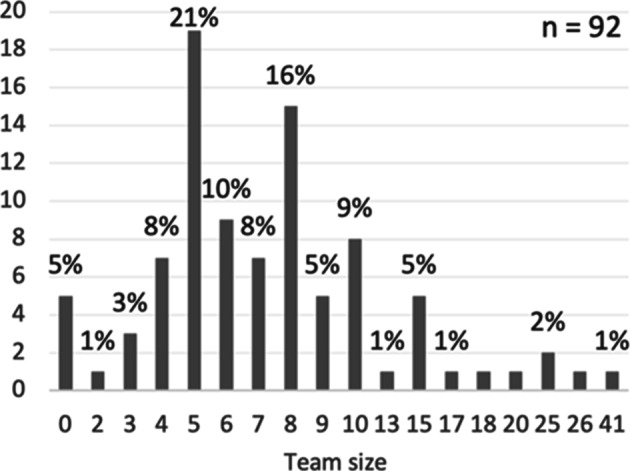
Team size. Values on the y-axis correspond to the frequency of respondents

**Fig. 7:**
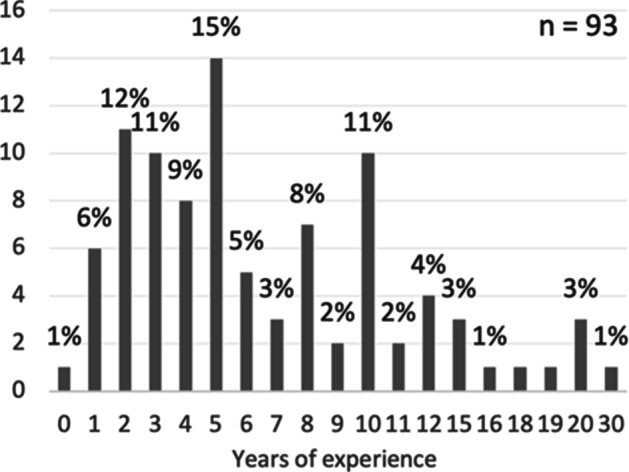
Years of experience in ASD. Values on the y-axis correspond to the frequency of respondents

Of the respondents who answered the questionnaire, 70% were male, 27% female, and 3% were either non-binary or did not want to specify. Most of the respondents (94%) were located in Europe while the remaining 6% were located in North America (3%) and Asia (3%). Looking into the different domains of the respondents, 43% worked in IT (e.g. software technology), 28% in embedded systems (e.g. automotive), and 29% in other domains (e.g. entertainment/media). The majority of the respondents (54%) had a technical role (e.g. developer), 26% had a management role (e.g. development manager), and 20% had an agile role (e.g. scrum master). The reported team sizes ranged from zero to 41 (as shown in Fig. 6), with an average team size of eight. The respondents experience of ASD ranged from zero to 30 years (as shown in Fig. 7) with an average of seven years.

Five percent of the respondents reported that they did not belong to a team, that is, a team size of zero. Most of the respondents were either part of exactly the same team (36%) or mostly the same team (27%) as before the pandemic, while 15% had changed team during the pandemic but still employed at the same company, and 21% had changed employment.

Before the pandemic, about half (48%) of the respondents never worked remotely, 45% worked mainly in office but sometimes remotely, 2% split the time evenly between working in office and remote, 3% mainly worked remotely but sometimes in the office, and only 2% worked remotely. At the time of the questionnaire, i.e. one year into the pandemic, a vast majority (78%) only worked remotely, 17% mainly worked remotely, 4% only worked in the office, and 1% worked as much in the office as remotely.

**Fig. 8:**
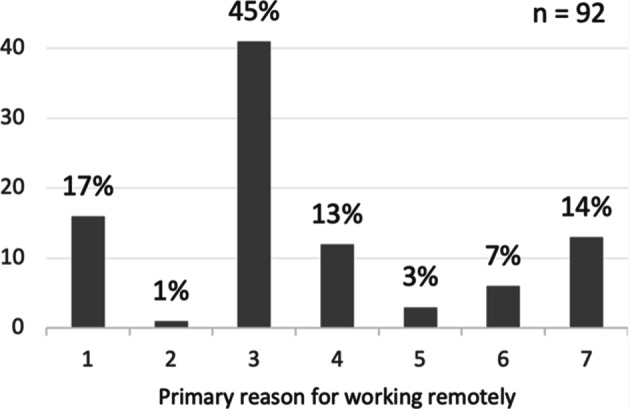
Primary reason for working remotely, where values 1-3 are due to recommendation from 1) company, 2) government, 3) company & government, and 4-6 due to enforcement by 4) company, 5) government, 6) company & government, and value 7 is own choice. Values on the y-axis correspond to the frequency of respondents

**Fig. 9:**
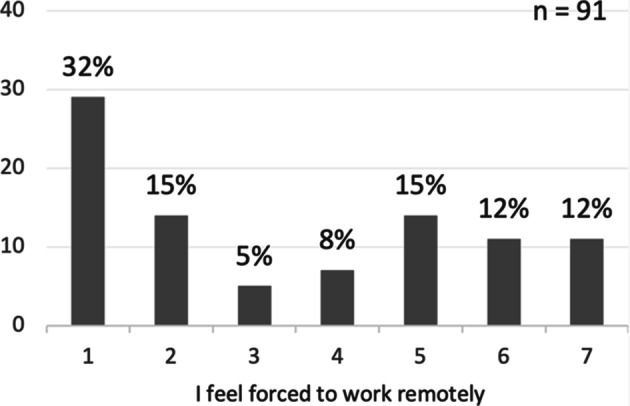
Whether feeling forced to work remotely. Where 1 is strongly disagree, 4 neither disagree nor agree, and 7 strongly agree. Values on the y-axis correspond to the frequency of respondents

Among the respondents who worked remotely at the time of answering the questionnaire, 63% stated that the primary reason for working remotely was due to a recommendation from the company and/or government, 23% stated it was due to an enforcement from the company and/or government, while 14% stated it was their own choice, as shown in Fig. 8. The majority (52%) of the respondents do not feel forced to work remotely, 39% felt forced, while the remaining 8% were neutral, as illustrated in Fig. 9.

### Analysis of Questionnaire

The Bayesian analysis resulted in a total of four significant predictors (i.e., predictors having a significant effect^8^), and 31 significant effects for 29 different outcomes. Significant effects are identified by either comparing the lower and upper credible interval (CI)^9^ values of population level effects, or by plotting probability densities, as seen in Fig. 13. An effect is significant if the CI values do not include zero, that is, both the lower and upper CI are either negative or positive (Furia et al. 2019). In other words, an effect is significant negative if both values are negative, and significant positive if both values are positive.

The most common significant predictor was whether a practitioner (i.e., respondents of the questionnaire) feels forced to work remotely, which was significant for 23 outcomes. The other three significant predictors were team constellation (significant for four outcomes), reason to work remotely (three outcomes) and role (one outcome). The predictors are referred hereafter to the abbreviations described in Table 4.

**Table 4:** Abbreviations for each significant predictor

Question ID	Name	Abbreviation
Q12	Feeling forced to work remotely or not	Forced
Q11	Recommendation, enforcement or own choice to work remotely	Reason
Q6	Role you mainly work in	Role
Q3	Same team or changed team or employment	Team Constellation

Below, the significant effects are presented in relation to the sections of the questionnaire, namely: agile practices, productivity and performance, well-being and work environment, meetings, communication, and teamwork

**Fig. 10:**
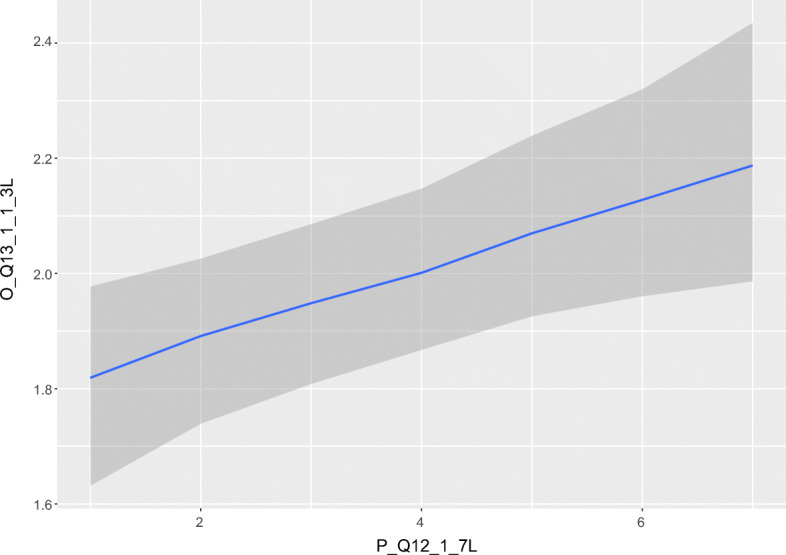
The graph illustrates how the predictor Forced (on x-axis) affects how challenging a stand up meeting/daily scrum is experienced (on y-axis). On the y-axis, y < 2 is *less challenging* and > 2 is *more challenging*. On the x-axis, x < 4 is *not feeling forced* and x > 4 is *feeling forced*. In other words, the more forced a practitioner feels to work remotely, the more challenging is a stand up meeting/daily scrum experienced

**Fig. 11:**
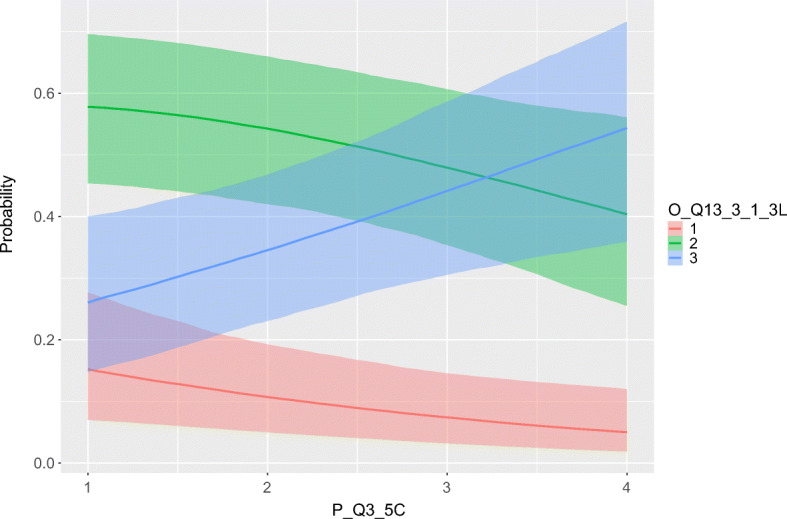
The graph illustrates how the predictor Team Constellation (on x-axis) affects the probability mass for how challenging sprint/iteration is experienced (on y-axis). The colors indicate the degree of challenge, where red is *less challenging*, green is *same as before*, and blue is *more challenging*. For example, the blue positive slope shows how the probability that a practitioner experiences sprint/iteration as more challenging increases as changes in team constellation increase

**Agile practices.**: Two predictors (Forced and Team Constellation) affect how challenging six agile practices (outcomes) are experienced now compared to before the COVID-19 pandemic, as shown in Table 5. The three agile practices stand up meeting/daily scrum, refactoring, and sustainable pace have Forced as a significant *positive* predictor, while sprint/iteration, sprint/iteration planning and sprint/iteration review/demo have Team Constellation as a significant *positive* predictor. This means, e.g., that stand up meeting/daily scrum is experienced to be *more* challenging now than compared to before the pandemic, if the respondent *feels forced* to work remotely. Similarly, if a practitioner has changed team or employment (Team Constellation) since the beginning of the COVID-19 pandemic, an agile practice such as sprint/iteration is experienced to be *more* challenging now. These effects are visualized in Figs. 10 and 11.

**Table 5:** Agile practices—outcomes having significant predictors

Question ID	Outcome	Significant predictor	Direction
Q13_1	Stand up meeting/ Daily Scrum	Forced	Positive
Q13_2	Sprint/Iteration	Team Constellation	Positive
Q13_3	Sprint/Iteration planning	Team Constellation	Positive
Q13_4	Sprint/Iteration review/demo	Team Constellation	Positive
Q13_15	Refactoring	Forced	Positive
Q13_19	Sustainable Pace	Forced	Positive

**Productivity and performance**.: The results of the Bayesian analysis show that six outcomes have Forced as a significant *negative* predictor, and Forced is a significant *positive* predictor for time spent on administrative work, as shown in Table 6. In addition, time spent on breaks has Reason as a significant *positive* predictor. This means, e.g., that productivity is experienced to be *lower* now than compared to before the pandemic, if the respondent *feels forced* to work remotely; and that the time spent on breaks is *higher* now than compared to before the pandemic, if the respondent works remotely due to own choice rather than due to recommendation. In addition, a practitioner who *does not feel forced* to work remotely experiences *less* distractions now than before the pandemic, compared to a practitioner who does *feel forced* to work remotely.

**Table 6:** Productivity and performance—the following outcomes had significant predictors regarding productivity aspects

Question ID	Outcome	Significant	Direction
		predictor	
Q16_1	On an average work day now, I feel... (productivity)	Forced	Negative
Q17_1	I focus better on my job now...	Forced	Negative
Q17_2	I believe there are less distractions now...	Forced	Negative
Q17_4	I believe the work I produce now is of higher quality...	Forced	Negative
Q17_5	I believe my team is more productive now...	Forced	Negative
Q17_6	I believe the work my team produces is of higher quality now...	Forced	Negative
Q18_10	Time spent on administrative work	Forced	Positive
Q18_13	Time spent on breaks	Reason	Positive

**Well-being and work environment**.: Feeling forced to work remotely has a significant *negative* effect on well-being, satisfaction of work environment, feeling of being appreciated, and motivation, as shown in Table 7. Meaning, a practitioner who feels forced to work remotely experiences a lower well-being, is less satisfied with the work environment, and feels less appreciated and motivated to work, than someone who does not feel forced to work remotely. The feeling of being overwhelmed by work differs from other outcomes as it has two significant predictors instead of one (see Table 7). It has Forced as a significant *positive* predictor and Reason as a significant *negative*. This means that a practitioner feels *more overwhelmed* by work if he/she *feels forced* to work remotely, and *less overwhelmed* if the practitioner works remotely due to *own choice* rather than *recommendation*.
**Meetings**.: As seen in Table 8, meeting quality, the extent to which meetings are face-to-face, brainstorming, and whether all people make themselves heard have Forced as a significant *negative* predictor, while it is a *positive* significant predictor for meeting frequency. Similar to the feeling of being overwhelmed by work, meeting quality differs from other outcomes as it has two significant predictors; both Forced and Team Constellation as significant *negative* predictors. This means that the meeting quality is experienced to be *lower* if a practitioner *feels forced* to work remotely and/or has *changed team or employment*.

**Table 7:** Well Being—the following outcomes had significant predictors regarding well-being and work environment

Question ID	Outcome	Significant predictor(s)	Direction
Q19_1	Well-being in general	Forced	Negative
Q19_4	Satisfaction of work environment	Forced	Negative
Q19_5	I feel appreciated	Forced	Negative
Q20_1	I am motivated to work	Forced	Negative
Q20_2	I am overwhelmed by work	Reason; Forced	Negative; Positive

**Table 8:** Meetings—the following outcomes had significant predictors regarding meeting aspects

Question ID	Outcome	Significant	Direction
		predictor(s)	
Q21_1	Meeting Quality	Forced; Team Const.	Negative; Negative
Q21_2	Meeting Frequency	Forced	Positive
Q21_7	Extent to which meetings are face-to-face (in person)	Forced	Negative
Q22_3	Brainstorming	Forced	Negative
Q22_10	All people make themselves heard	Forced	Negative

**Fig. 12:**
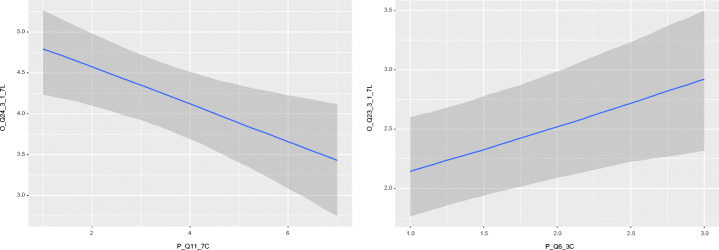
Conditional effects for the predictors Reason (Q11) and Role (Q6) for to what extent practitioners spend time on communication and how often they communicate in person, respectively. Predictors are plotted on the x-axis and outcomes on the y-axis. The effect in the graph to the left is condensed between *increased (5)* and *decreased (3)* of the outcome, whereas the effect in the graph to the right is condensed below *same as before (4)*. The latter means that practitioners clearly have experienced to communicate in person less often now

**Communication**.: Table 9 shows the outcomes related to communication and their respective significant effects. Communication quality and how often practitioners use written communication have Forced as a significant *negative* and *positive* predictor respectively. In addition, how often practitioners communicate in person has Role as a significant *positive* predictor whereas the extent to which respondents spend time on communication has Reason as a significant *negative* predictor. This means that, written communication is used *more often* and that the communication quality is *lower* now than compared to before than pandemic, if the practitioner *feels forced* to work remotely. As seen to the right in Fig. 12, all types of roles have experienced a decrease in how often they communicate in person, but a practitioner with a *non-technical* role experienced a *smaller decrease* than a practitioner with a *technical* role. Use of written communication has clearly increased for most practitioners, but practitioners who *feel forced* to work remotely has experienced a *larger increase* than those who *do not feel forced*. Furthermore, a practitioner spends *less* time on communication now, compared to before the pandemic, if working remotely due to own choice rather than due to recommendation, as seen to the left in Fig. 12.

**Fig. 13:**
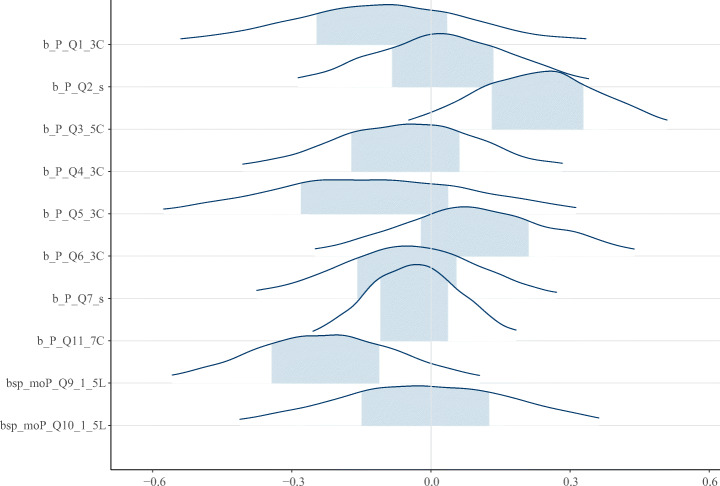
When analyzing Forced as an outcome, the extent to which a practitioner worked remotely or in office before the pandemic (Q9, second from bottom) and especially their Team Constellation (Q3, third from top) show strong tendencies to whether a practitioner feels forced or not

**Teamwork**.: For teamwork, there was only one outcome with a significant predictor, namely team morale which has Forced as a significant *negative* predictor. Meaning, the experienced team morale in a team has decreased compared to before the pandemic, if the practitioner feels forced to work remotely.
**Investigating forced as an outcome**.: As mentioned in Section 3.1.4, besides analyzing the Forced aspect as a predictor, it was also analyzed as an outcome dependent on the other predictors. Looking at Fig. 13, it is evident that to what extent a practitioner worked remotely or in office before the pandemic (Q9) and especially their Team Constellation (Q3) show strong tendencies to whether a practitioner feels forced or not, but they are not significant. For example, a practitioner who does not feel forced may be part of exactly the same team as before the pandemic whereas someone who does feel forced may have changed team or employment.

**Table 9:** Communication—the following outcomes had significant predictors regarding communication aspects

Question ID	Outcome	Significant predictor	Direction
Q23_2	I use written communication	Forced	Positive
Q23_3	I communicate in person	Role	Positive
Q24_1	Communication Quality	Forced	Negative
Q24_3	Time (I spend on communication)	Reason	Negative

### Employment of Agile Development and Ways of Working (RQ1)

**Fig. 14:**
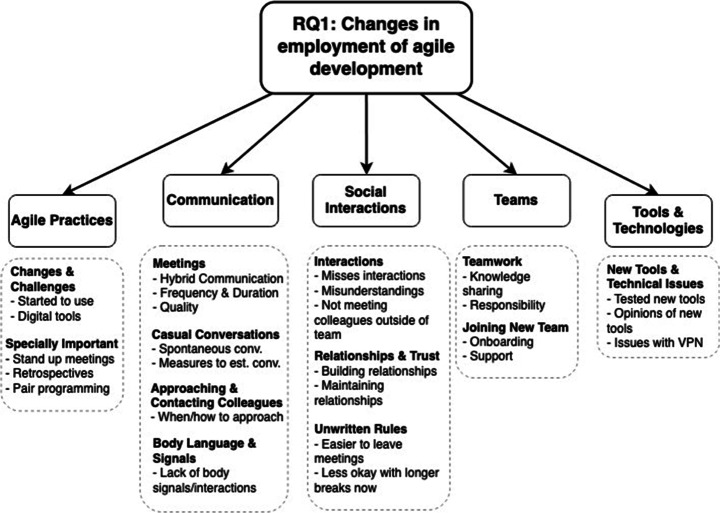
A detailed view of the areas that are connected to the employment of ASD and ways of working

Looking at how industry practitioners^10^ employ ASD, and how their ways of working have changed due to the COVID-19 pandemic (RQ1), 12 sub-areas (e.g., specially important) were identified and divided into five areas (e.g., agile practices), as illustrated in Fig. 14. The identified areas are discussed below.

#### Agile Practices

**Fig. 15:**
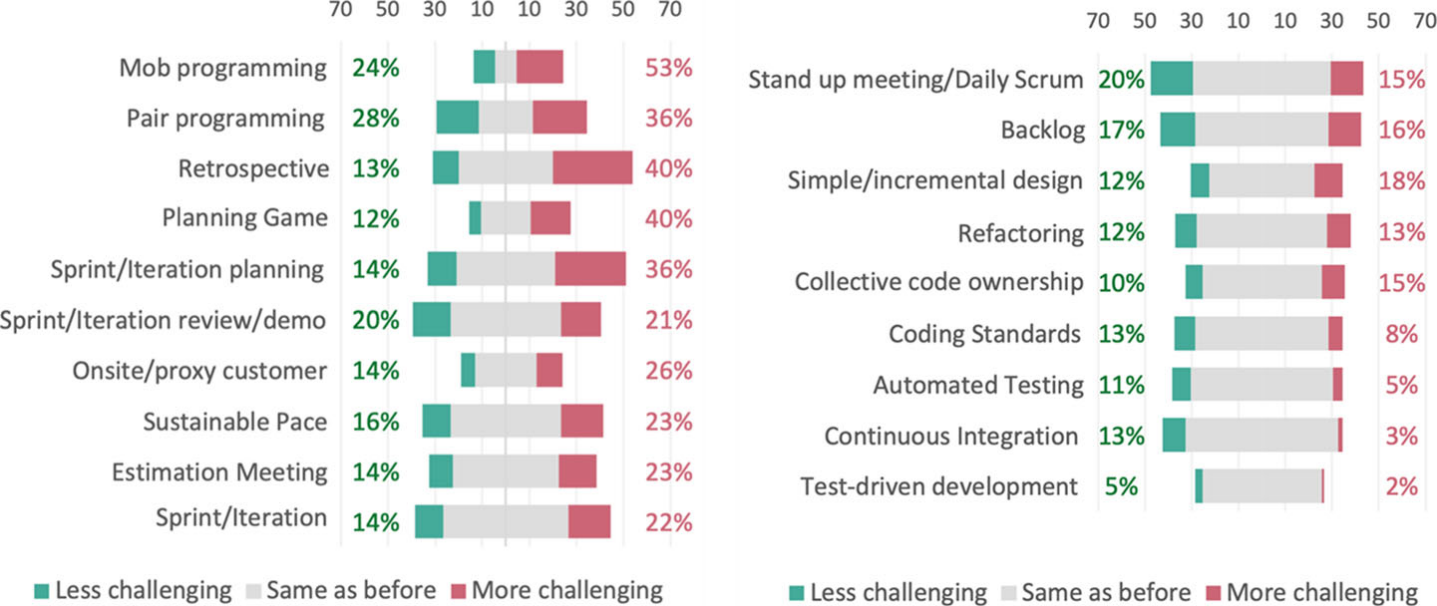
*Q13: How challenging do you find the following practices now, compared to before the pandemic?* All 19 agile practices sorted in a descending order of percentage of changes reported by the respondents, where percentage of change equals percentage of participants who reported a practice to be less challenging (green percentage) plus percentage of participants who reported a practice to be more challenging (red percentage). The agile practice with the highest percentage of change is in the top left corner (Mob programming) while the practice with the lowest percentage of change is in the bottom right (Test-driven development)

Overall, in 14 out of 19 agile practices, a majority of the respondents experienced the practices to be as challenging now as before the pandemic, as illustrated in Fig. 15. In four practices (mob programming, pair programming, retrospective and planning game), a majority of the respondents reported a change in how challenging the practices were experienced, while for the agile practice sprint/iteration planning, 50% of the respondents reported a change and 50% reported reported no change in how challenging the practice was perceived. Looking at Fig. 15, we can see that agile practices of more social nature, such as as mob programming and retrospective, indicated a higher percentage of changes than practices of more technical nature, such as continuous integration and test-driven development.

##### Changes & Challenges in Agile Practices

Three agile practices in particular were mentioned both in the questionnaire and during interviews regarding changes and challenges, namely retrospectives, pair programming, and stand up meetings.

Regarding *retrospectives*, the results from the questionnaire show that retrospectives have become *more challenging* for many practitioners during the COVID-19 pandemic. Several interviewees mentioned that the lack of body language and signals, and that people talk over each other and do not open up during retrospectives are reasons that have contributed to why retrospectives are perceived as more challenging now than before the pandemic. The importance of using video cameras during retrospectives to convey body language was mentioned by several respondents. One respondent explained, “*always use video for body language, social interaction and human connection*”. The content in retrospectives have changed, they do not only discuss ways of working, but also how people are doing and COVID-19 related topics. One respondent explained, “*retro has always been important, but now it is a place to vent, talk about how we are doing, and do some mental health support*”.

*Pair programming* is one of the agile practices that differs the most from other practices as there is a relatively even distribution of respondents across the three different alternatives: less challenging, same as before, and more challenging. Based on the interviews, it is evident that several practitioners have started to use, or extended their use of pair programming. One interviewee described that teams have started to use pair programming more seriously during the COVID-19 pandemic. There are two reasons for using pair programming, for knowledge sharing and to socialize. Regarding knowledge sharing, one interviewee mentioned that pair programming has been the rescue for new team members during the pandemic. Regarding social aspects, one respondent described that “*we are pair-programming a lot more now than before the pandemic. I guess this is because we do not see each other as much as before, so pair programming enables us to see and hear each other, and collaborate together on tasks, which are all aspects of team interactions that have taken a hit because of the pandemic.*”.

Although *stand up meeting/daily scrum* was not the most challenging practice (as shown in Fig. 15), changes have been made to stand up meeting/daily scrum during the COVID-19 pandemic. Respondents reported that the frequency of stand ups is almost the same as before, but with a little change towards an increase. Several interviewees reported on adding more daily stand ups, e.g one more after lunch, because there has been a lack of natural syncing during the day. One respondent described that “*since spontaneous communication is a lot more difficult when working remotely we now have two stand ups per day*”. Some practitioners described that they are fine with longer stand ups since there are less interactions and spontaneous conversations when working remotely. One respondent explained, “*daily Scrum meetings have become slightly longer but the team agrees this is OK because we do not see each other during the day like we did at the office*”. Another respondent described that they have longer daily scrums since “*you perceive less what the others are doing*”.

##### Specially Important

A common theme that emerged is how some agile practices have been more important than others during the COVID-19 pandemic. As the daily contact and interaction with colleagues have been strongly reduced during the COVID-19 pandemic, practitioners have especially used daily stand ups, retrospectives, and pair programming to address the lack of interaction.

*Daily stand up* has been an important agile practice to keep the interaction alive during the COVID-19 pandemic, and not only as a function to update each other on work progress, but also as a way to maintain the feeling of working in a team and to check in on how people are doing. Before the pandemic, practitioners would see each other outside of the daily stand ups, but when working remotely, the daily stand up is one of the few moments where practitioners actually see and talk to each other. One respondent explained, “*daily standup is the most important. [...] And since attendance is mandatory people _actually_ communicate in a different way than they would without the standup. Moreover, the standup increases the feeling of being part of a team since there is always a little chit chat and joking around in those meetings*”.

Several practitioners stated that *retrospectives* are particularly important now, compared to before the pandemic, as it has become more difficult to communicate and pick up on how people are doing when not sitting next to each other. One respondents explained that in the office, “*it was easier to pick up issues that people had but when everyone is remote, it is much harder to see if someone is not happy with something or not feeling well etc.*”. Another respondent mentioned that it is particularly important as they do not “*sit together physically any more, which makes the retrospectives to be so much more important for the team to work as good as possible*”.

*Pair programming* was reported to be a specially important agile practice during the COVID-19 pandemic by several practitioners. Reasons were, social interaction, teamwork, knowledge sharing, and quality. For knowledge sharing and socially wise, pair programming has been a rescue for new team members. One respondent described how pair programming is more important now “*since the normal social interactions have been strongly reduced (i.e. not working in the same room anymore)*”. Another respondent explained that distributed pair programming has been important for quality and “*without this practice, quality suffers and it takes longer to deliver stories.*”.

#### Communication

It is not surprising that practitioners communicate less often in person and have less face-to-face communication. Instead, there is an increase in written and digital communication. However, there are challenges with digital communication, e.g., a decrease of body language and signals. One respondent mentioned, when communicating digitally, it is difficult “*to transmit the same amount of information via the different digital solutions, such as Skype, Teams, etc.*”. In addition to challenges with digital communication, the practitioners experienced challenges with hybrid communication, where some work in office and some at home, as it creates an uneven balance. Several interviewees stated that hybrid communication does not work as well as when everyone is onsite or remote. One respondent experienced that a mix of people working in the office and at home has not worked well, and that he/she ‘*personally think that remote teams works if all is working remotely*”.

In general, informal conversations have decreased, while work related conversations have increased during the COVID-19 pandemic. In addition, communication with people outside of the practitioners’ own teams has decreased, and practitioners only talk to people from other teams if they really need to work with them.

**Fig. 16:**
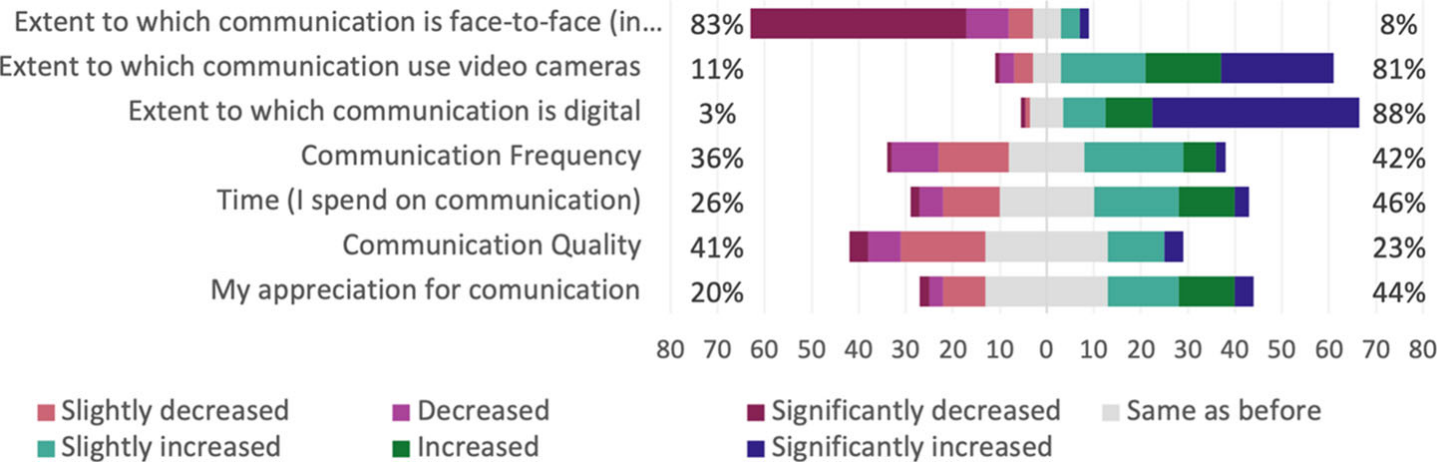
Q24: Compared to before the pandemic, how would you rate the following aspects regarding the communication you have now? Sorted in ascending order of percentage of *same as before*

Regarding the transition to digital communication, communication quality have decreased since the beginning of the COVID-19 pandemic, whereas appreciation for communication has increased, as seen in Fig. 16. However, one respondent mentioned that communication “*has improved, being more concise, to the point and in overall warmer, but more of a human touch.*”.

##### Meetings

**Fig. 17:**
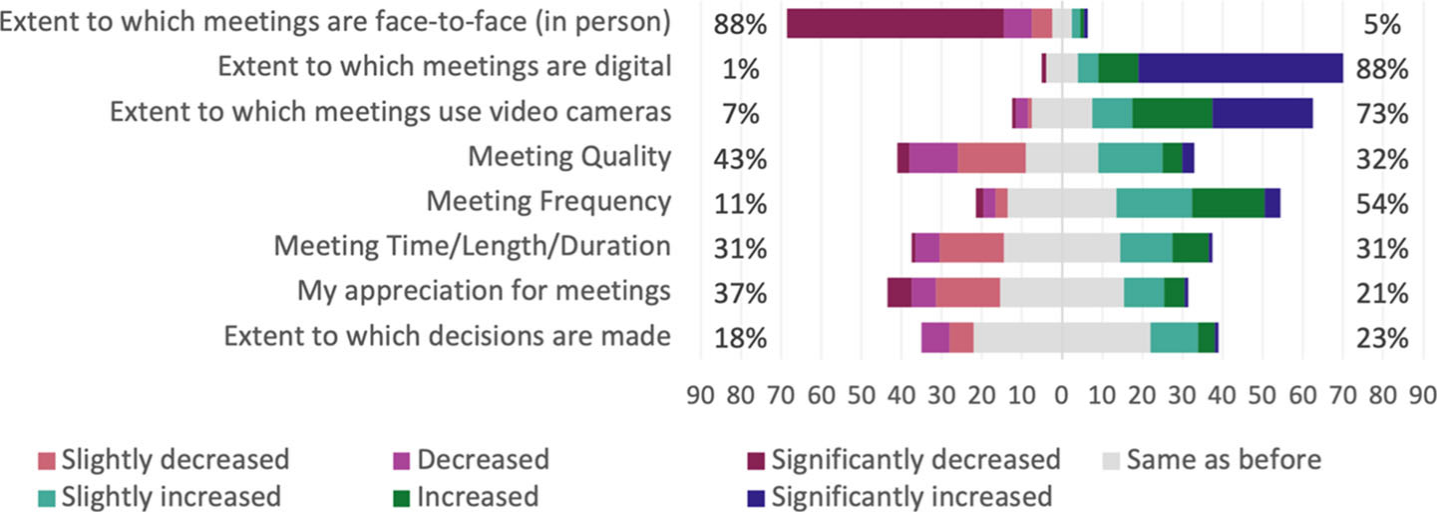
Q21: Compared to before the pandemic, how would you rate the following aspects regarding the meetings you have now? Sorted in ascending order of percentage of *same as before*

There have been changes to meetings due to the transition to remote work, e.g., a significant decrease of meetings that are face to face, and a significant increase of to what extent meetings are digital and use video cameras, as illustrated in Fig. 17. A majority of the practitioners have experienced a change in meeting quality, where some believe it has increased and others that it has decreased. One respondent mentioned that the “*meeting discipline (clear agenda, note taking, formal decisions) have improved tremendously.*”. On the other hand, several interviewees mentioned that people often talk over each other or that they are too silent in digital meetings, which contributes to a decreased quality. In addition, there is a significant decrease in people who make themselves heard during meetings. Two interviewees confirmed this by explaining that some of their colleagues are very silent during meetings, which was not the case before the COVID-19 pandemic. One of the interviewees further explained that digital meetings “*probably brings out the worst in people, allowing them to be more introverted*”.

A majority of the respondents experienced an increase of meetings, which is both confirmed and contradicted by the interviewees. Two interviewees expressed that their and other colleagues’ schedules are packed, and that they experienced “*back to back*” meetings because of the digital meeting structure. Moreover, several respondents mentioned that they have meetings almost every day to ensure that people are up to speed and feeling less lonely. In contrast, one interviewee believed that he/she attends fewer meetings now, compared to before the pandemic, which may be because he/she actively tries not to attend meetings now and that it is easier to leave meetings when they are digital.

##### Casual Conversations

Casual conversations that used to be carried out spontaneously in hallways and before meetings have been reduced. One reason is that it is not possible, in a remote setting, to glance or overhear what other people say or do. Thus, enabling casual conversations is more difficult now. The lack of casual conversations does not only affect work, but also people’s social life and well-being. One interviewee (P4), who works as an agile coach, mentioned a consequence of the lack of casual conversations. This have affected the work of agile coaches who are more focused on organization and leadership, as much of their work is to talk to managers by the coffee machines to make changes. P4 mentioned that it is not as flexible to book a meeting for these casual conversations. Since there has been a loss of casual conversations, measures to reinforce similar types of conversations have taken place, e.g., digital tea or coffee breaks, lunches, catch-ups and text channels, with the aim to “*ensure staying close as a team*”, as explained by one respondent.

##### Body Language & Signals

Practitioners have experienced a significant decrease in how often they communicate in person and to what extent they communicate face-to-face. Thus, the value of body language, signals, and facial expressions has become evident. Several interviewees mentioned that, in face-to-face communication, you can read each other’s body language, voice and facial expressions, which makes resolving design issues and other types of problems a lot easier.

##### Approaching & Contacting Colleagues

There have been changes to how practitioners’ approach and contact their colleagues during the COVID-19 pandemic. Personal preference and lack of unwritten rules influence how practitioners approach their colleagues. Instead of approaching colleagues in the office, written communication or phone calls are used. However, practitioners are more reluctant to contact a colleague directly without thinking it through first, which was not the case before the COVID-19 pandemic. One respondent explained that “*there is a lot less rubber ducking and you don’t ask people for help or about stuff before you have thought it through thoroughly. Both positives and negatives with that.*”, while another respondent said that “*people think once more before disturbing and this helps with focusing on the given task and not being disturbed.*”.

#### Social Interactions

A general theme is the lack of, and importance of having social interactions with colleagues. Most interactions have been replaced by digital social engagement activities such as digital coffee breaks, which have been experienced mostly as good alternatives, but not necessarily substitutes for having them in person.

##### Interactions

Interaction with colleagues, both within and outside of the own team, either physically or digitally, is important to build relationships, solve problems, well-being, and to avoid misunderstandings. Practitioners miss the social nature of being around other people and the spontaneous conversations it may induce. As one interviewee explained, he/she “*really miss the water cooler moments at work in particular, because you often bump in to each other in the kitchen and be like, I am working on this, how would you do it? You get some really cool interactions with that*”. Misunderstandings in digital meetings and chats are examples of bad effects of digital communication since it is not possible to send a feeling in the same way as in real life, as explained by one interviewee. The interviewee has experienced misunderstandings while chatting and learned that it is better to talk than to write. To avoid misunderstandings in chats and written communication, one respondent explained that “*clear communication is very important, and keeping things organized in text form. I feel that extra care has to be taken when explaining things in text since you can’t immediately catch misunderstandings*”. In addition, as explained by several practitioners, it is important that people know each other personally to avoid misunderstandings, but also for opening up to colleagues and sharing things with each other.

##### Relationships & Trust

About half of the respondents reported a decrease in team morale, and for team inclusion and trust, there were almost as many reporting an increase as a decrease, compared to before the COVID-19 pandemic. One reason why some respondents reported an increase in team morale was that everyone has been exposed to the same situation. The results reveal that it has become more difficult to create personal relationships when working remotely. However, it is not impossible, it may just take longer time to establish, and with fewer people. In order to maintain relationships and trust, it is important to have social interactions and non-work-related casual conversations.

##### Unwritten Rules

When working remotely, practitioners are not necessarily expected to behave in the same way as they did in the office. It may be easier to deviate from the unwritten rules that existed in a physical setting, e.g., it is easier to drop out of irrelevant meetings when working remotely, compared to when working in the office. One interviewee elaborated by comparing remote meetings to physical ones, “*so, before if you were stuck into a physical meeting, you cannot just drop in ‘oh, I am not actually interested in this’ and then walk out, it would be more awkward you know.*”. On the other hand, there are new unwritten rules to follow in a remote setting, e.g., related to breaks. In general, practitioners felt expected to attend a coffee break in the office, but do not feel expected to do so when working remotely. One interviewee described that all people do not join coffee breaks anymore. In the office it was more of an expectation to join, but now when they are working remotely, it is an optional activity that is not necessary to join.

#### Teams

**Fig. 18:**
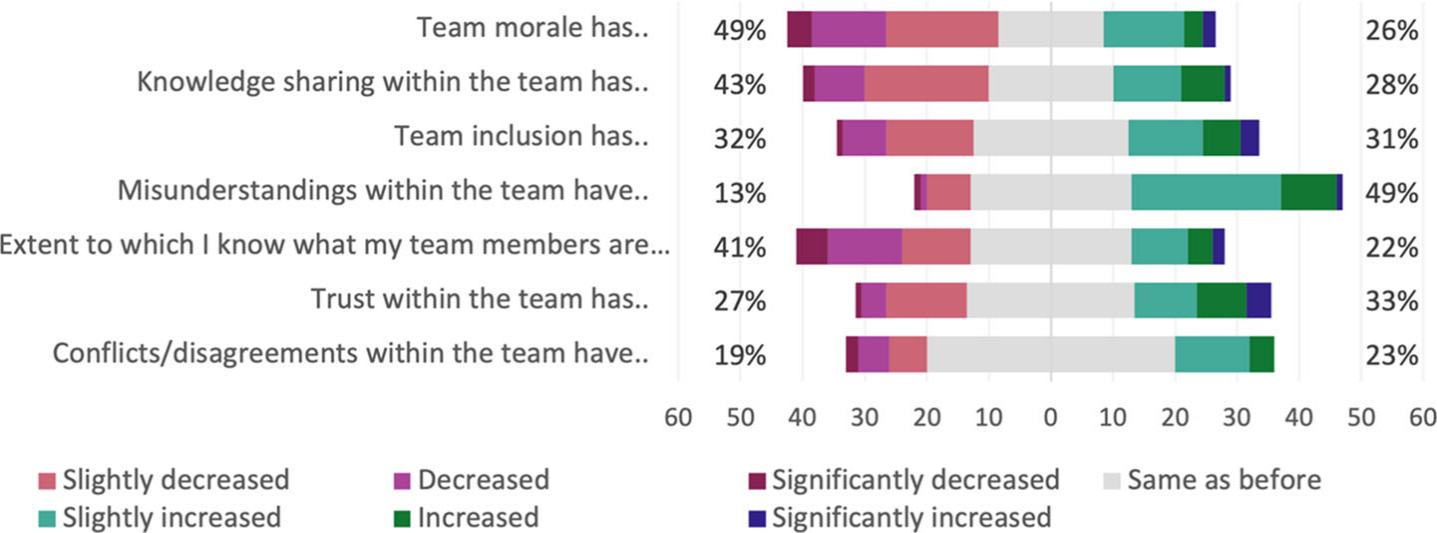
Q26: Compared to before the pandemic, how would you rate the following aspects regarding your team now? Sorted in ascending order of percentage of *same as before*

Compared to before the pandemic, several respondents experienced a decrease in team morale, knowledge sharing, and to what extent they know what their team members are working on, as shown in Fig. 18. About half of the respondents (49%) have experienced an increase of misunderstandings, but regarding conflicts and disagreements, a majority did not report any increase or decrease.

##### Teamwork

Several practitioners described that pair programming has been an effective approach for knowledge sharing, and thus reduce the need for syncing. Moreover, practitioners started to use pair programming during the COVID-19 pandemic to create knowledge sharing and this has been a rescue for new team members. One reason for a decreased team morale was related to work that still needs to be done in the office, e.g., working with hardware. One interviewee explained, in the beginning of the COVID-19 pandemic, it was a bit unclear who was responsible for what. The team members who still worked in office ended up with higher workload than those who worked remotely and became irritated on those who did not come to the office.

##### Joining New Team

Many practitioners joined a new team during the pandemic; 35% of the respondents, and 71% of the interviewees. There are challenges when joining a new team during a pandemic; company feeling, getting to know colleagues, and avoiding misunderstandings. Getting support from the company and colleagues is important, in particular in the beginning when, e.g., processes are unknown. In addition, when joining a new team while working remotely, getting support from colleagues, e.g., by being able to ask questions, is important for adapting to the situation. Another type of support, which was explained by one interviewee, is that the company encouraged employees to take a day off when feeling stressed, which was taken more seriously now compared to before the pandemic. Almost a majority of the respondents reported that they have as much support from their company now as they had before the pandemic, but for 21% it had decreased compared to 34% for which it had increased.

Interviewee P6, who joined a new company during the pandemic, experienced it to be challenging to work from home in the beginning of the pandemic and that he prefers face-to-face communication since it is easier to interpret signals that way, compared to when working remotely. Similarly, a respondent raised how it is necessary to reduce the gap between new and old team members, the respondent explained, “*people that is new at the company doesn’t have the same team feeling that old members have. We have to work on that!*”. From a manager perspective, P2 explained that it is a challenge for managers to build teams remotely, and that managers who are responsible for building teams have to think about “*how do I do that remotely?*”.

#### Tools & Technologies

Despite being a necessary and somewhat forced decision to experiment with new tools, the usage has provided practitioners with insights on how tools can both facilitate and hinder their work, and how it is possible to work remotely with agile development. Some tools were already in use prior the COVID-19 pandemic, e.g., issue tracking tools, but the usage has become more serious during the pandemic. In the beginning of the COVID-19 pandemic, many practitioners experienced technical issues as the infrastructure was not in place to deal with everyone working remotely at once. However, most issues seem to have been resolved.

##### New Tools & Technical Issues

As a result of the transition to digital communication, many practitioners have tried new tools, and increased the extent to which they use video cameras and video conferencing tools. The experiences of the new tools vary, some are positive while others are negative. For example, physical whiteboards and other materials have been replaced by digital ones. One respondent explained, “*digital whiteboards like Miro and Mural is really awesome and it would have been hard to cooperate and do what we do without them. They are really a life saver in our work*”. One interviewee described that they replaced all physical materials with digital boards like Miro and Mural, which worked well, even for keeping up the interaction. Similarly, some interviewees described that they have converted from physical post-it notes to digital tools. This was a positive experience since everyone can actually see all the notes, as explained by one interviewee. However, digital tools do not always provide an equally positive experience. For example, people talking over each other, microphone problems, and people who tends to look at the presentation instead of the people despite using video cameras are all examples of issues with digital communication. One interviewee also described that it is more difficult to screen share something quickly compared to drawing something on a physical whiteboard.

### Impact of Recommended or Enforced Remote Work (RQ2)

**Fig. 19:**
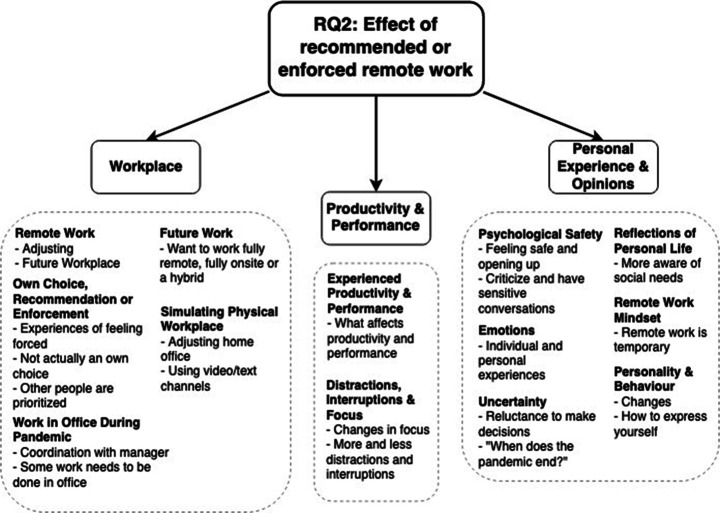
A detailed view of the areas that are connected to how practitioners have been affected by recommended and/or enforced remote work

In analyzing RQ2, how practitioners have been affected by recommended and/or enforced remote work due to social restrictions, certain areas have had more prominent impacts than others: Workplace, Productivity & Performance, and Personal Experience & Opinions, as illustrated in Fig. 19.

#### Workplace

Five areas related to workplace were identified: remote work; own choice, recommendation or enforcement to work remotely; work in office during pandemic; future work; and simulating physical workplace.

##### Remote Work

Most practitioners had limited experience of working remotely prior to the COVID-19 pandemic, and thus needed to adjust to the new situation. However, after one year of remote work, the practitioners have become more positive towards remote work and are now used to the situation. One interviewee explained how remote work was a new experience, and that the biggest challenge was to adapt to the changes of not working in office anymore; however, by taking it step by step it went well. Overall, practitioners have learned a lot by the fact that almost everyone is working remotely and that everyone has faced the same situation. One respondent explained, “*we’ve learned a lot and especially if everyone is put up for the same challenges. Then it works a lot better since everyone needs to adapt, not just a few. [...] If some individuals are not keen on adapting and don’t want to learn. Then it is really hard.*”. From the questionnaire, the results reveal that there is a mix of opinions regarding the work environment, 38% of the respondents were less satisfied with their work environment whereas 31% were more satisfied. This was also reflected by the interviewees, some prefers to continue to work remotely, whereas others do not.

Among the most positive consequences of remote work that were communicated during the interviews were, the benefits of not having to commute, and the perceived flexibility and freedom of working remotely. Several interviewees perceived having more flexibility and freedom since they can work from any place they want and manage their time more easily when working remotely. For example, the ability to work on tasks at any time they want. This flexibility is also reflected by the questionnaire results, which show that about half (45%) of the respondents reported an increase of work-life balance, whereas 28% reported a decrease. However, some practitioners were worried that not meeting each other in person will have negative consequences in the long run. The lack of interaction may affect peoples’ feelings and personalities, as well as the ability to gain advantages of working as a team.

##### Own Choice, Recommendation or Enforcement to Work Remotely

A majority of the respondents stated that they work remotely due to a recommendation from company and/or government, about 25% due to enforcement and the rest work remotely due to their own choice. Among the interviewees, six out of seven stated that they have been recommended to work from home. A majority of the practitioners do not feel forced to work remotely; however, 39% of the respondents do.

Several interviewees explained the feeling of being forced to work remotely. P6 mentioned that he felt forced to work from home as there were no capabilities to work from home in the beginning, while P2 felt that the transition to remote work was in a forced direction. However, P1 looked at it from a different perspective, stating that “*if we were forced to do something, that would be to work from the office, and not the other way around*”, but if you did not feel comfortable doing so, it was not an issue, he added.

Several interviewees expressed that they did not feel forced to work remotely because they were allowed to go into office if they needed. Although P7 did not felt forced, he stated that in practice they were forced because of the lockdown. Another perspective was mentioned by P3 who explained that it is important not to force people to work in office, especially the ones who either belong to a risk group themselves or live with someone who does. In some situations, the decision to work from home or in the office was not the individuals’ own choice “per se”, they just followed the protocol. For example, several interviewees mentioned that they were told to follow their clients’ recommendations regarding working from home or not, or that their companies had communicated that the ones who could work from home should do that to allow others—who could not work from home—to work in the office. This aspect of putting others first was a recurring theme throughout the interviews.

##### Work in Office During Pandemic

The questionnaire results show a significant increase in the number of people who now work from home the majority of the time. However, some still work as much remotely as in office, and some work the majority of the time in office. Most of the interviewees mentioned that some work still needs to be done in office. For example, working in a department with certain services that need to be handled in office, troubleshooting hardware, or picking up work-related things such as canvases and whiteboards, are all reasons to still be and work in the office. One respondent explained why they still need to work in the office, “*recently we’ve frequently had to have a local team member go physically to the office to re-start machines that failed on applying Windows updates*”. For some, one impact of the COVID-19 pandemic is that hardware testing is done less frequent due to working from home. However, in some cases, as for P1, the responsibility for working with the hardware can be taken care of by the ones who still go to the office.

##### Future Work

All interviewees reflected about future work situations, especially for when the COVID-19 pandemic is over. The majority want to work in a hybrid model, if possible. A hybrid model, according to the interviewees, includes working in the office some days and at home the other days of the week. Reasons for working in a hybrid model were, being able to leave the home a couple of days a week, and due to missing the office and social interactions. One interviewee explained that he/she preferred a hybrid model and preferred that the whole team went to the office the same days. Although the majority of the interviewees prefers work in a hybrid model, there are a few exceptions, and some have colleagues or team members that feel differently. Some people think it is boring to work from home and others prefer to work in office as they have family members who would disturb them at home.

Several interviewees discussed the future use of office space. One view was that offices may serve a more specific value rather than being occupied by empty desks. One interviewee mentioned the possibility for changing certain rooms to workshop rooms where teams can meet the days they all are in the office. Similarly, another view concerned, in particular, having some days a week dedicated to certain teams.

##### Simulating Physical Workplace

Based on the interviews, the results show that several processes and tools, such as having core work hours or using open video and audio channels, are used to simulate a physical workplace. One way to simulate the feeling of a physical workplace, which several interviewees have either heard of or tried themselves, is to have an open video call or voice chat that anyone can connect to during the day. This was also mentioned by a respondent who described it as a “team radio”, “*an always on call that makes it feel somewhat like being at the office*”. An informal channel where “*people can come and go as they please*”, and where other teams can join in case they need help or if there is an urgent issue going on. Besides an open video call or voice chat, one interviewee mentioned that they have tried Wonder.me to simulate a physical workplace.

#### Personal Experience & Opinions

The interviews show that personal experiences and opinions regarding both work-related and social aspects have changed since the beginning of the COVID-19 pandemic. A lack of psychological safety and spontaneous meetings have resulted in practitioners not opening up to, or sharing as much with their colleagues as they did before the COVID-19 pandemic.

##### Psychological Safety

The interviewees explained how feelings regarding psychological safety in remote settings have become more important during the COVID-19 pandemic, even though a majority of the respondents have experienced the same amount of surveillance from their companies and colleagues now, as before the pandemic. Based on the interviews, it became clear that some people feel less safe in online meetings, and that without psychological safety, some discussions will not be raised.

When critical things need to be discussed, some people find it dangerous to do this in writing or in a chat. Instead, it should be discussed in online/physical meetings. The fear of leaving a digital trace in writing may lead to that several things are put on hold. This is especially true regarding certain meetings and discussions that are seen as safer to have in a physical location, that is, discussions where conflicts are expected to arise and people to have more feelings. One interviewee explained how some people are reluctant to write down certain things due to a fear of leaving digital traces in text, and that some people do not want to voice opinions about other colleagues and their behaviour in written and/or oral digital communication. The interviewee explained that such a discussion is a type of conversation that you would have during informal face-to-face communication, and that it is important to have it since “*you might also understand if you have done something wrong yourself.*”.

The questionnaire results show that practitioners do not make themselves heard as often now as compared to before the COVID-19 pandemic. The interviewees confirm that they have experienced that some colleagues are very silent during meetings now, and that there is a lack of people opening up to other colleagues. Several reasons for this behaviour were mentioned by the interviewees, e.g., fear of leaving digital traces, lack of spontaneous meetings, and lack of knowing each other personally. Another reason, which was mentioned by P7, is due to the way meetings are conducted now, and that it “*probably brings out the worst in people, allowing them to be more introverted*”.

##### Emotions

A majority of the respondents reported that they feel as appreciated as before the COVID-19 pandemic and that their work is as meaningful as before. However, P6 mentioned that depression and emotional problems have increased during the pandemic, “*there is no doubt that the Corona has affected peoples’ personalities and feelings*”. Another interviewee explained that it is harder to keep up the motivation when working from home, compared to when working in the office, and one reason is the lack of variation.

##### Uncertainty

The interviewees described how the COVID-19 pandemic has been a time of uncertainties regarding how to work remotely, an uncertainty that creates a reluctance to make decisions, and an uncertainty of when the COVID-19 pandemic is over. One interviewee mentioned that “*nobody expected that this pandemic to take this so long time*” while another one wondered “*in a month or so, are people vaccinated by then, or will this go on for 5 years?*”. This has led to that certain types of decisions and discussions were postponed insinuating that they would be resurrected when the COVID-19 pandemic has ended. One interviewee explained, “*it is very hard to make any bigger decisions when there is so much uncertainty*”, and since the beginning of the pandemic, the whole situation has been marked by it.

##### Reflections of Personal Life

Some of the interviewees reflected on their personal life and needs, and how they believe that more people are aware of their social needs now, and in general, that people take better care of themselves mentally now.

##### Remote Work Mindset

Several interviewees expressed a mindset that the remote work is only temporary, and that they soon will be back in the office. Some of the interviewees held on to this belief for a long time and have even put things on hold because of it. For example, in P7’s case, everyone has known that remote work is only temporary, since it has been the plan all along to go back to the office when allowed.

##### Personality & Behaviour

The interviews show that there has been a change in practitioners’ behaviour and personalities. One interviewee explained how he has become less strict regarding daily stand ups and its content. Now, the interviewee is more lenient with people not being on point when they explain what they have done and will do. Before the COVID-19 pandemic, the interviewee would ask people to have sync discussions after the stand up, but now people can be more verbose and have more discussions.

Another change in behaviour is regarding coffee breaks. In some cases, people who used to turn up for physical coffee breaks do not join the digital ones. One reason is that people do not think it is important since they have been in digital meetings with same people all day long. In contrast, one respondent described how they have forced digital coffee breaks “*to ensure staying close as a team*”.

The interviews show that people have reflected on how they prefer to communicate, some prefer to text while others prefer to talk. However, it is important that people are able to express themselves correctly to avoid misunderstandings. How to communicate, in writing or talking, depends on the individual preferences and the content. One interviewee explained that he/she prefers to communicate in text as long as the text is not too long. In contrast, another interviewee mentioned, “*100% better in expressing myself via talking*”. One reason for preferring talking is the experience of misunderstandings when chatting with colleagues. The type of communication may differ depending on the content. As one interviewee described, if it is of more technical character, writing is preferred, while if it is about a bug, talking is preferred.

One interviewee expressed how online meetings bring out the worst in people, allowing them to be more introverted. However, the interviewee also brought up that colleagues who—before the pandemic—used to be quiet and not hang around people, now try to join discussions more often. Changes in personality were explicitly mentioned by two interviewees, where both stated that their personality has changed since the beginning of the COVID-19 pandemic. P6 described himself to be a very extroverted person before the pandemic, but now due to the COVID-19 pandemic and the lack of communication with colleagues, his personality has changed towards being more introverted. P6 explained, you get more and more introverted, step by step, not within one week or one month, but over time. The interaction between people and society has been cut, and thus may damage peoples’ personalities. Contrary to P6, P7 described himself to be introverted one year ago, but now he has become more extroverted.

#### Productivity & Performance

The results show that there have been changes regarding productivity and performance. While the questionnaire results suggest a clear increase in productivity, the interviews reveal how productivity may fluctuate and that it depends on the situation. In addition, there has been a considerable decrease of interruptions and distractions.

##### Experienced Productivity & Performance

**Fig. 20:**
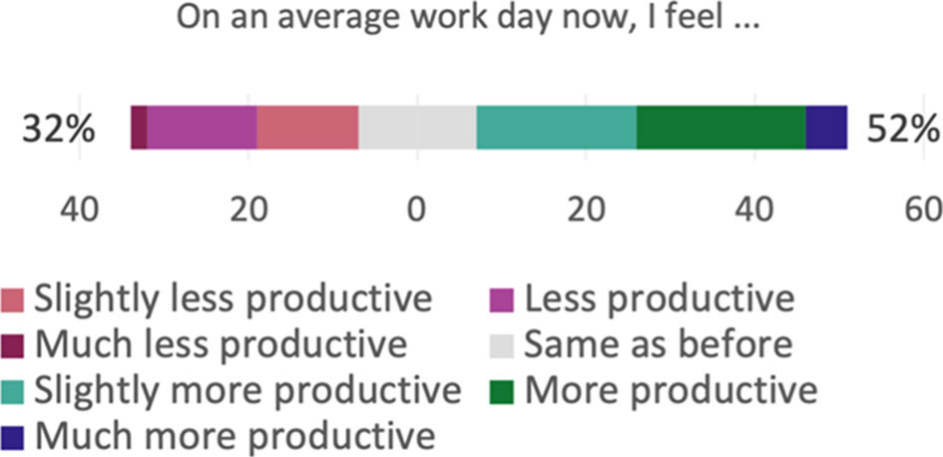
Self-reported productivity

**Fig. 21:**
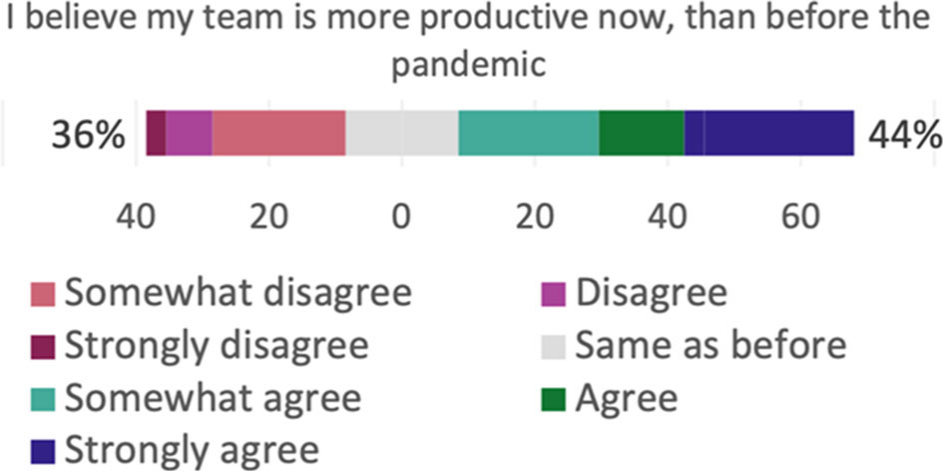
Team productivity

A majority (52%) of the respondents believe that their productivity, on an average work day, has increased since the beginning of the COVID-19 pandemic, while about a third believe that their productivity has decreased, as shown in Fig. 20. The results for team productivity are similar, as shown in Fig. 21.

Several interviewees described how their productivity and performance have remained the same or increased, but for two of them, the productivity has been fluctuating. One interviewee believed that the productivity and performance were the same as before the pandemic, but there is no evidence for this. However, the interviewee mentioned that initially—in the beginning of the COVID-19 pandemic—the productivity went up, but later on into the pandemic it went downhill. One reason was distractions of non-work-related activities. Another interviewee explained that there are several things that affect the productivity, e.g., emotions and personal life. When the interviewee was happy and had a good work-life balance, the productivity went up. However, lack of interactions and social activities, not only with colleagues but also with friends and family had a negative impact on productivity.

**Fig. 22:**
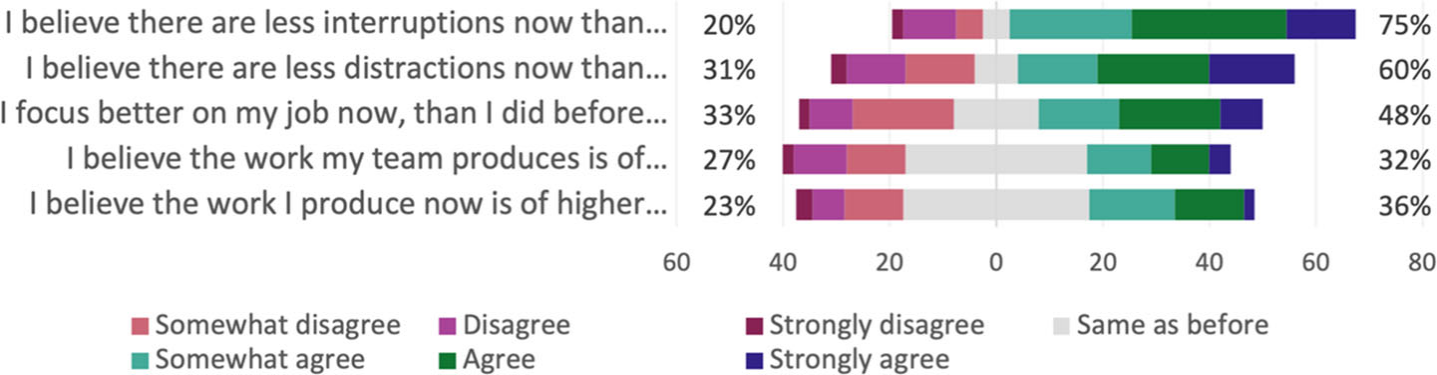
Q17: Please fill in to what extent you agree with the following statements (regarding Productivity & Performance). Sorted in ascending order of percentage of *same as before*

Regarding whether the respondents’ reported quality of work is higher now, the results are relatively evenly distributed across disagreement, agreement and same as before, as seen in Fig. 22. One of the respondents described that there are several factors that contribute to general improvement: “*no time is wasted for commuting; number, length and frequency of meetings decreased, while their impact increased; [...] team members and the team as a whole increased its productivity and efficiency; sense of creativity, sense of independence, and thus our motivation also increased. All this results in improving our performance, work related satisfaction and quality of life.*” Another respondent was not as equally positive and stated that a “*remote team can work, even though I personally believe that working as team at an office is better for productivity*”.

##### Distractions, Interruptions & Focus

Figure 22 shows that a vast majority of the respondents have experienced less distractions and interruptions now, compared to before the COVID-19 pandemic. However, among the interviewees there were different opinions. As one interviewee explained, there are fewer interruptions and organic discussions now, as you cannot simply turn around and ask someone about something. Another interviewee agreed that the old distractions and interruptions have disappeared, but they have been replaced by new ones. The interviewee explained, there are more distractions regarding, .e.g., Slack notifications, so “*it comes out even*”. Other new distractions are types of distractions at home, such as partners and children.

About half (48%) of the respondents believe that they focus better on their job now, compared to before the COVID-19 pandemic, whereas 33% disagree. A majority of the respondents believe that they are expected to be as responsive and available, or more now, compared to before the COVID-19 pandemic. From the interviews it became clear that some have experienced a better focus. One interviewee explained, before the COVID-19 pandemic, the interviewee would not always stay and fix an issue at the end of the day, but now, the interviewee can as he/she does not have to leave work to go home. However, there may be negative consequences of the individuals increased job focus. One interviewee mentioned that a possible consequence of having a better focus when working from home may be that people work more individually now than before the COVID-19 pandemic. Similarly, one respondent mentioned that the team is“*much more personal focused now. It really takes hard steering to get the team to work as a team and not as individuals*”.

## Discussion

In this section, the results are discussed and related to the literature. Section 5.1 discuss general findings. Section 5.2 discuss the first research question, while the second research question is discussed in Section 5.3.

### General Discussion of Results

Table 10 provides a summary of the key findings and practical implications in general.

**Table 10:** Summary of key findings and implications in general

Finding	Implication
Lack of social interactions	Lack of social interactions can result in negative consequences in relation
	to psychological safety, well-being, and teamwork in general.
Informal environments	Informal environments are important for non-work-related conversations
	and social interactions, both within and outside the team, which have a
	positive impact on work-related activities.
Team maturity and	Lack of social activities and informal environments, and communication
teamwork debt	issues, may have led to a debt regarding team maturity and teamwork
	that needs to be handled when the teams are back onsite again.

Several different areas of ASD have been affected by the COVID-19 pandemic. The most affected areas are related to social interactions and communication, whereas more technical areas have been less affected. It is evident that technical agile practices, such as test-driven development and continuous integration, and other aspects of a more individual character have been functioning well, even during the COVID-19 pandemic. However, for aspects of more social nature, the results are different. The lack of social interactions can result in negative consequences in relation to, for example, psychological safety, personalities, well-being and teamwork in general. Looking at the agile values and principles of the agile manifesto, to value “individuals and interactions over processes and tools” (Beck et al. 2001a), have become more difficult now since there is a need to put more emphasis on processes and tools in a remote setting. Practitioners cannot communicate without relying on digital tools or setting up processes for how to communicate and interact with colleagues. Although digital communication works, it is not the same as face-to-face communication.

Agile principles such as “build projects around motivated individuals” and “give them the environment and support they need” (Beck et al. 2001a), have become more difficult to follow and address, which is in line with (Mancl and Fraser 2020). The practitioners have indeed received the needed technical support, but lacked support for creating the needed informal environments. The informal environments are important for having non-work-related conversations and social interactions, both within and outside of their own teams, which have a positive impact on work-related activities. However, this does not happen as often now as before the COVID-19 pandemic. Some practitioners feel inhibited by the virtual nature of communication, and experience that activities of these kinds are more challenging now compared to before the COVID-19 pandemic. However, despite this, many practitioners do believe that remote work has worked better than expected, and realized that there are some positive aspects of remote work, such as the flexibility of managing their own time.

Some practitioners expressed that they would like to work remotely, at least some days a week, even after the COVID-19 pandemic. This is particularly interesting, considering the lack of social interactions when working remotely and the belief that communication works best if all are onsite or all are remote, whereas hybrid communication^11^ is not preferred. What the long-term effects and implications of this will be, are yet to be shown. However, there is a risk that practitioners have been affected in a way that will make it challenging to go back to what previously was known as the normal, even if possible. Hence, it is important that these challenges are addressed and mitigated. Therefore, companies should expect a debt regarding team maturity and teamwork that needs to be taken action upon when teams once again are back onsite.

### Employment of Agile Development and Ways of Working (RQ1)

Table 11 provides a summary of the key findings and practical implications for employment of agile development and ways of working.

**Table 11:** Summary of key findings and implications for employment of agile development and ways of working

Finding	Implication
Decreased appreciation	There has been a significant increase in number of meetings since work-related
for meetings in	and catching up with colleagues are scheduled as meetings, and an increase in
general	back-to-back meetings which may result in more stressed and less motivated
	practitioners. The increase in meetings in combination with challenges with
	digital meeting structure, e.g., hybrid meetings, have led to a decreased
	appreciation for meetings.
Hybrid meetings	Hybrid meetings are less effective and more challenging compared to meetings
	where all participants are either remote or onsite. Hybrid meetings creates a
	gap between participants that are onsite and those who are remote.
Stand up meetings,	These three agile practices have
retrospectives, and	been particularly important. They all have been used to compensate for lack
pair programming	of casual conversations. Syncing conversations that usually existed in person
	in the office are now carried out during more and longer stand up meetings.
	etrospective aspects, such as reflections and continuous improvement have
	become more important than the retrospective meetings per se. Pair
	programming is not only particularly important for teams and teamwork in
	a remote setting, but also for integrating new team members and facilitate
	knowledge sharing.

The transition from in person to digital communication has led to a significant increase of meetings, which confirm the results in Miller et al. (2021). However, Russo et al. (2021b) reported on a decrease in number of meetings during the COVID-19 pandemic. The difference between the studies may be explained by the focus and timing, i.e., Russo et al. (2021b) mainly focused on technical roles, such as developers, and their study was conducted a few months into the pandemic, while we focused on more than just technical roles (e.g., agile coaches, product owners etc.) and our study was conducted one year into the pandemic.

One reason for the increased number of meetings is that not only work-related topics, but also catching up with colleagues and asking them how they are doing are scheduled as meetings, which is in line with Neumann et al. (2021). Hence, all communication, including what used to be brought up in spontaneous and casual conversations, have now turned into meetings. However, it is not only an increase in frequency of meetings, but also an increase of back-to-back meetings, which in turn may result in more stressed and less motivated practitioners. The increase of meetings, in combination with the challenges with the digital meeting structure, e.g., hybrid meetings, may explain why the appreciation for meetings have decreased now, compared to before the COVID-19 pandemic. However, non-scheduled activities with fewer attendees, e.g., pair programming, are seen as more spontaneous, casual, and easier to conduct than digital meetings. This may explain why the appreciation for communication has increased, while the appreciation for meetings has decreased.

Hybrid meetings are seen to be less effective and more challenging compared to meetings where all participants are either remote or onsite, which is in line with (Marek et al. 2021; Mancl and Fraser 2020; McConnell and Stuart 2020). One reason hybrid meetings are less effective is because it creates a gap between participants that are onsite and those who are remote. This was only realized when everyone started to work remotely. The gap refers to how the meeting participants interact with each other. The ones who are onsite can easily talk to each other in a spontaneous way, while the ones who are remote have to rely on digital tools, and therefore have to approach and contact their colleagues in a more deliberate way. This unbalance creates a difference that not only can affect the relationships between team members, but may also result in that some information is lost since only the onsite participants are involved in the informal interactions that keep people up to date on, e.g., what to discuss in an upcoming meeting or regarding who is working when and from where. However, despite the challenges of hybrid communication, practitioners still want to work in a hybrid model in the future. This creates a challenge regarding future work arrangements and workplaces since the challenges of hybrid communication will still be evident. How to work in a hybrid model, and to maintain social interactions, keep hybrid communication to a minimum, and being able to reap some of the benefits of remote work is yet to be seen.

The importance of having casual conversations has become evident and there have been attempts to create a virtual environment for casual conversations by integrating them into three agile practices, namely: stand up meetings, retrospectives, and pair programming. These three agile practices have been particularly important during the COVID-19 pandemic since it is in relation to these practices that casual conversations can occur more naturally now. Schmidtner et al. (2021) found that only daily stand up meetings are used to address the lack of casual conversations, and that stand up meetings and retrospectives were found to be particularly important. One reason that may explain the difference between the studies is the respondents. A majority of the respondents in Schmidtner et al. (2021) had management positions, while in our study, a majority had technical roles. Meaning, respondents with management positions may not do pair programming as much as, e.g., developers, and therefore not view pair programming to be especially important.

To compensate for the lack of casual conversations, practitioners have been increasing the number of stand ups, extending the use of pair programming, and valuing retrospectives more now, than before the COVID-19 pandemic. Both non-work-related and work-related conversations that took place in the office are now manifested in the increased use of remote pair programming to enable closer teamwork. In addition, pair programming is not only particularly important for teams and teamwork in a remote setting, but also for integrating new team members and facilitate knowledge sharing during a pandemic. Syncing conversations that usually existed in hallways, by the coffee machine, in the office kitchen, or before meetings, are now carried out during more and longer stand up meetings. Retrospective aspects, such as reflections and continuous improvement have become more important than the retrospective meetings per se. In addition, retrospectives have become a space to ask how people are feeling and doing during the COVID-19 pandemic, and the importance of retrospectives is confirmed by Miller et al. (2021).

Mancl and Fraser (2020) discussed that “agile work practices are harder to perform given the virtual nature of meetings and interactions”, compared to before the COVID-19 pandemic, which is partly confirmed in our study. Some agile practices, e.g., retrospectives and pair programming, are more challenging, some, e.g., continuous integration, automated testing are neither more nor less challenging, while other agile practices, e.g., stand ups, are less challenging to perform now. In general, agile practices of more social nature (e.g., pair/mob programming) have been experienced to be more challenging than those of a more technical nature (e.g., test-driven development). One reason is that online tools were already in use for several technical practices before the COVID-19 pandemic; for example, Jira was used for issue tracking before the pandemic, and is still in use. However, for pair/mob programming, there has been an increased usage, both among experienced and inexperienced users, for which the latter may had to deal with a learning curve. This may explain why pair/mob programming is more challenging to perform now, especially since there has been a challenge in testing and using new online tools for digital pair programming.

Contrary to other social agile practices, stand up meetings were less challenging to perform now, compared to before the pandemic. The reason seems to be that there is a difference between stand up meetings and general meetings, both regarding the structure and appreciation. A stand up meeting is used for syncing between team members in a sequential way. Team members taking turns is also discussed in Mancl and Fraser (2020) as being more suitable in a virtual setting than in a physical one. The challenges of a digital meeting structure, such as people talking over each other and that some are silent, may therefore not be as prominent in a virtual stand up meeting as in a virtual retrospective or general virtual meeting. This may also explain why stand up meetings are more appreciated than meetings in general.

### Impact of Recommended or Enforced Remote Work (RQ2)

Table 12 provides a summary of the key findings and practical implications for recommended or enforced remote work.

**Table 12:** Summary of key findings and implications for recommended or enforced remote work

Finding	Implication
Feeling forced	There is a difference between being and feeling forced to work remotely, which
	is important to acknowledge in order to minimize its effect. Feeling forced affects
	many aspects negatively, such as productivity, communication, meeting quality, and
	well-being. This is essential when considering future work arrangements. It is
	important to either let the practitioner choose where to work (in office or remote),
	or all team members should be working under the same premise, i.e., all in office or
	all remotely.
Conflicts and	A substantial number of practitioners have changed teams during the pandemic, but
disagreements	conflicts and disagreements in their teams have not substantially increased. For a
	new team to mature, and eventually perform as a group, it is important to go through
	a phase of conflicts and disagreements. This may be a warning signal that the teams
	have not matured enough to perform at their very best. Hence, more conflicts may
	arise when the teams start working together onsite.

This study is unique in the way that it investigates what impact a forced or recommended remote work situation has on agile software development and its practitioners. Other studies (McConnell and Stuart 2020; Machado et al. 2021; Oz and Crooks 2020) have mentioned the forced and/or mandatory situation, but not investigated the feelings of being forced to work remotely further.

One of the main findings of this study is whether a practitioner feels forced to work remotely or not, has an impact on e.g., their well-being, productivity, communication, and experience of stand up meetings. Even though there is a considerable difference in *being* forced, and *feeling* forced to work remotely, the difference is not acknowledged. For example, several practitioners mentioned that it is not possible to feel forced to work remotely since they are not forced to do so, and in practice, they can work from wherever they want. However, there is an underlying theme of putting the needs and wishes of other colleagues first, which may create a feeling of being forced to work remotely. Even though a practitioner would rather work in the office, he/she may feel the need to work from home since other colleagues may not be able to create an optimal work environment at home. Another way of feeling forced is in the opposite direction, i.e., the experience of feeling forced work in the office when working from home is preferred. For example, practitioners who are not as worried of catching the COVID-19 virus as their colleagues, may feel forced to go into the office when there are, e.g., hardware issues that requires an onsite presence.

Practitioners who have expressed feeling forced to work remotely have been told to work remotely, while practitioners who are free to go into office when needed do not feel forced. This may mean that they have to be forced to in order to feel forced. However, feeling forced to work remotely or not is not dependent on the primary reason for working from home, i.e., whether it is a recommendation, enforcement or own choice. It rather seems to depend on switching teams and/or employment during the COVID-19 pandemic, and that practitioners who have not met their new team members in real life, are more negative to the work situation, and therefore also may feel more forced to work remotely than others.

It is important to acknowledge that there indeed is a difference between *being* and *feeling* forced to work remotely in order to minimize its effect on agile software development aspects. Especially in similar circumstances in the future since feeling forced affects many aspects negatively, such as productivity, communication, meeting quality, and well-being. This is essential when considering future work arrangements. If a practitioner has the opportunity to choose where to work, he/she should be able to do so without taking other colleagues wishes and needs into consideration. On the other hand, if a practitioner cannot choose, he/she and his/her colleagues should be working under the same premise, that is, all in office or all remotely.

A majority of the practitioners have experienced an increased productivity or not been affected negatively, which is in line with NicCanna et al. (2021), Bao et al. (2020), Ford et al. (2020), but contradicts Ralph et al. (2020). However, almost a third reported a decreased productivity, which contributes to the fact that practitioners have dichotomous experiences of productivity, as discussed in Ford et al. (2020). Unique to this study is the analysis of the relation between productivity and feeling forced to work remotely. Although, not necessarily surprising, one of the main findings is that a practitioner who feels forced to work remotely experiences a lower productivity now, compared to before the COVID-19 pandemic, than a practitioner who does not feel forced.

One unexpected finding was the large number of practitioners who switched teams and/or employment during the COVID-19 pandemic. Despite not being the focus of this study, several aspects regarding the challenges of joining a new team or company during the pandemic became evident, which is in line with reported virtual on-boarding in Kude (2020), Mancl and Fraser (2020). In addition, knowing your colleagues, and especially having met them in real life, have a big impact on opening up, avoiding misunderstandings, working better as a team in general, and being more content with your work situation. If possible, new team members should meet their new colleagues in person, e.g., via social on-boarding activities, which is in line with Rizvi et al. (2015). However, completely new teams formed during the COVID-19 pandemic do not experience the same challenges, which may be due to that everyone has been exposed to the same challenge and/or developed a better understanding for each other’s situation.

Another interesting finding, which is also discussed in Neumann et al. (2021), is that conflicts and disagreements in teams have not substantially increased now, compared to before the COVID-19 pandemic. This is interesting since a substantial number of practitioners have changed teams during the pandemic. That conflicts and disagreements have not increased can partly be explained by a reluctance to bringing up sensitive conversations digitally. Another explanation may be related to the belief that remote work is only temporary, and thus these conversations have been put on hold until they are back in the office. Considering the challenges with digital communication, and the large number of practitioners who have changed teams during the COVID-19 pandemic, it would not have been surprising if conflicts and disagreements had increased. As first introduced by Tuckman (1965), a new group usually go through four phases—Forming, Storming, Norming and Performing—until they can perform at their very best. Storming is a phase where many conflicts within a group appear, and it is important to go through this phase in order to mature, and eventually perform as a group (Tuckman 1965). However, since conflicts and disagreements in teams have not substantially increased, this may be a warning signal that several teams have not gone through all of these phases, which means that they have not matured enough to perform at their very best. In addition, this may lead to that more conflicts and issues arise when the teams start working together onsite again. To make room for, and address a team maturing dept, as also suggested by Kude (2020), is of out most importance even now, but especially when teams do go back the office.

## Threats to Validity

To avoid potential threats that may arise when using an explanatory sequential design, we addressed the recommendations by (Creswell and Creswell 2017). Findings could be compromised and invalidated if all quantitative results have not been considered before deciding on what data to follow up (Creswell and Creswell 2017). We addressed these threats by not only looking into the significant results from the Bayesian analysis of the questionnaire, but also by looking at the non-significant results as well as the answers to the open questions before designing the interview protocol.

One threat is a poor questionnaire design which may lead to a misunderstanding of the intent and content of the topic (Wohlin et al. 2000). Although it is not possible to exclude the threat of misunderstanding the questions, we minimized these threats by conducting two iterations of pilot studies. Both iterations collected feedback regarding the clarity and content of the questions in the questionnaire and was addressed and adjusted accordingly. As the majority of the questions of the questionnaire were of a seven point Likert-item format, it is possible that respondents did not notice minor changes in the scales, e.g., changing the range from decrease/increase to less often/more often. To minimize this risk, the scales were formatted in the same direction, i.e., left as negative and right as positive. Another threat that may affect how respondents answered is that they answer based on a preconceived knowledge of the aim of the study (Juristo and Moreno 2001). By only providing general information about the aim of the study, this threat was minimized.

Both regarding the questionnaire and the interviews, there is a risk that practitioners may not share their opinions or answer truthfully (Baskerville and Lee 1999), and thus making the results biased. To minimize this risk, we guaranteed complete anonymity and confidentiality as to all information divulged during the questionnaire and interviews. Moreover, keeping the interview sessions to 45 min, which was possible by collecting background information before the interview sessions started, alleviates maturation threats (Juristo and Moreno 2001).

Threat to selection bias is always present when study subjects are not fully randomly sampled. However, random sampling in Software Engineering research is rare (Amir and Ralph 2018; Baltes and Ralph 2020) since there are no credible lists of the entire population of, e.g., software projects, software practitioners, organizations, or teams (Amir and Ralph 2018). Therefore, for the questionnaire, we combined convenience, maximum variation, and snowball sampling to advertise the questionnaire in our industrial network and in industrial collaboration networks and communities dedicated to agile software development. For the interviews we used convenience sampling. This allowed us to collect data from practitioners with different backgrounds and roles from different companies. However, since most of the survey respondents were from Europe, and all but one interviewee were from Sweden, we do not claim that our results are representative of the views of all agile software development practitioners and companies in general.

Incorrect data (Robson 2002) is a threat to all studies of empirical nature, including this study. To reduce this threat, the audio recordings from the interviews together with the opportunity to validate the answers and interpretations of the answers with the participants lessening the risks of misunderstandings. In order to improve the reliability (Robson 2002) of the interviews, three steps were taken. First, the first two authors were present at each interview, which increases the reliability and reduces the risk of single researcher bias. Second, the interviews were conducted with different software developing companies, and each interview was performed in one session. Third, an interview protocol was used to make sure that all aspects were covered in each interview.

## Conclusion

In conclusion, an explanatory sequential mixed methods study was conducted to investigate how the involuntary shift to remote work and how social restrictions imposed by the COVID-19 pandemic have affected ASD, and how agile practitioners have been affected in terms of ways of working. Data were collected through a questionnaire with 96 respondents and in-depth semi-structured interviews with seven practitioners from seven different companies.

In general, the results reveal that the COVID-19 pandemic’s impact on ASD confirms the importance of the agile value “individuals and interactions over processes and tools”. Technical processes and tools work as well in a remote setting as in physical one, but without physical social interactions, practitioners find it difficult to work together in an efficient way. However, despite the challenges imposed by the COVID-19 pandemic, there is a positive attitude towards remote work, and working in a hybrid model in the future is preferred.

The findings for RQ1, how employment of ASD and ways of working have changed due to the COVID-19 pandemic, show that the most prominent aspects concern three agile practices, in particular, and the adjustment to digital communication. Stand up meeting, retrospective, and pair programming have been three especially important agile practices during the COVID-19 pandemic since these have been used to address the lack of social interaction. Stand up meeting has been one of the few moments when practitioners do see and talk to each other. Retrospectives have served as time and place to discuss well-being, both professionally and mentally, and pair programming has been used to both socialize and for knowledge sharing. Pair programming was also seen as especially important for new team members. The transition to digital communication has resulted in a lower communication quality and a reflection on what and when to communicate and approach colleagues. The remote work mindset in combination with less psychological safety and new team constellations during the COVID-19 pandemic, may lead to major consequences once back in office. As conflicts have not increased, despite a substantial increase of misunderstandings, there is a risk that the teams have not matured during the COVID-19 pandemic and that the discussions that they have postponed will explode when they are back in office.

The recommended and enforced remote work due to social restrictions have affected practitioners in many different ways (RQ2). However, it is not the reason per se that has had a major impact, but rather the aspect of feeling forced to work remotely. The findings show that feeling forced to work remotely has had a significant impact on, e.g., productivity, well-being, communication, and meetings. A lower productivity, well-being, and communication quality, and a higher meeting frequency are all examples of significant effects for a practitioner who feels forced to work remotely.

The results from this study show that the personality and behaviour of practitioners have changed since the beginning of the COVID-19 pandemic. Therefore, it would be interesting to study what different needs different personalities have regarding their workplace, both socially and physically. Furthermore, the results reveal challenges of joining a new team with social restrictions during the COVID-19 pandemic. It has become evident that there is a need for practitioners to have guidelines for how to build virtual teams effectively, both with and without social restrictions. Thus, future research should look into how to build virtual teams effectively, and how to address the needs of psychological safety in order to give the practitioners the environment they need virtually.

## Data Availability

R code for Bayesian analysis available at: 10.5281/zenodo.5115661

## Footnotes

1 http://www.questionpro.com

2 10.5281/zenodo.5115661

3 For a Likert-item, as described by Bürkner and Vuorre (2019) “ordered verbal (or numerical) labels are used to obtain discrete responses about a continuous psychological variable.”.

4 Standardizing the predictors “means to first center the variable and then divide it by its standard deviation” McElreath (2015, p. 111).

5 Normal(0, 0.25) corresponds to a normal distribution with a mean of 0 and a standard deviation of 0.25

6 10.5281/zenodo.5115661

7 https://www.qsrinternational.com/nvivo-qualitative-data-analysis-software/home

8 A significant effect means that one can be confident that the predictor has an impact on the outcome or, that there is a difference between different values of each predictor.

9 The CI reflects the uncertainty interval and is referred to as confidence intervals in frequentist statistics (Furia et al. 2019)

10 Practitioners refer to both interviewees and respondents from the questionnaire. A respondent refers to a respondent of the questionnaire, whereas an interviewee is either referred to by ID, for example, P1, or solely as interviewee.

11 Hybrid communication is when a part of attendees of a meeting or a conversation are onsite, and the other part are remote.
